# Inhibition of Small-Conductance Calcium-Activated Potassium Current (*I*_K,Ca_) Leads to Differential Atrial Electrophysiological Effects in a Horse Model of Persistent Atrial Fibrillation

**DOI:** 10.3389/fphys.2021.614483

**Published:** 2021-02-09

**Authors:** Merle Friederike Fenner, Giulia Gatta, Stefan Sattler, Marion Kuiper, Eva Melis Hesselkilde, Ditte M. T. Adler, Morten Smerup, Ulrich Schotten, Ulrik Sørensen, Jonas Goldin Diness, Thomas Jespersen, Sander Verheule, Arne Van Hunnik, Rikke Buhl

**Affiliations:** ^1^Department of Veterinary Clinical Sciences, Faculty of Health and Medical Sciences, University of Copenhagen, Taastrup, Denmark; ^2^Department of Physiology, Cardiovascular Research Institute Maastricht, Maastricht, Netherlands; ^3^Department of Biomedical Sciences, Faculty of Health and Medical Sciences, University of Copenhagen, Copenhagen, Denmark; ^4^Department of Cardiothoracic Surgery, The Heart Centre, Copenhagen University Hospital, Copenhagen, Denmark; ^5^Acesion Pharma ApS, Copenhagen, Denmark

**Keywords:** persistent atrial fibrillation, atrial selectivity, SK/K_Ca_2 channels, NS8593, epicardial contact mapping, AF conduction, inter-atrial heterogeneity, equine/horse model

## Abstract

**Background:**

Small-conductance Ca^2+^-activated K^+^ (K_Ca_2) channels have been proposed as a possible atrial-selective target to pharmacologically terminate atrial fibrillation (AF) and to maintain sinus rhythm. However, it has been hypothesized that the importance of the K_Ca_2 current—and thereby the efficacy of small-conductance Ca^2+^-activated K^+^ current (*I*_K,Ca_) inhibition—might be negatively related to AF duration and the extent of AF-induced remodeling.

**Experimental Approach and Methods:**

To address the hypothesis of the efficacy of *I*_K,Ca_ inhibition being dependent on AF duration, the anti-arrhythmic properties of the *I*_K,Ca_ inhibitor NS8593 (5 mg/kg) and its influence on atrial conduction were studied using epicardial high-density contact mapping in horses with persistent AF. Eleven Standardbred mares with tachypacing-induced persistent AF (42 ± 5 days of AF) were studied in an open-chest experiment. Unipolar AF electrograms were recorded and isochronal high-density maps analyzed to allow for the reconstruction of wave patterns and changes in electrophysiological parameters, such as atrial conduction velocity and AF cycle length. Atrial anti-arrhythmic properties and adverse effects of NS8593 on ventricular electrophysiology were evaluated by continuous surface ECG monitoring.

**Results:**

*I*_K,Ca_ inhibition by NS8593 administered intravenously had divergent effects on right and left AF complexity and propagation properties in this equine model of persistent AF. Despite global prolongation of AF cycle length, a slowing of conduction in the right atrium led to increased anisotropy and electrical dissociation, thus increasing AF complexity. In contrast, there was no significant change in AF complexity in the LA, and cardioversion of AF was not achieved.

**Conclusions:**

Intra-atrial heterogeneity in response to *I*_K,Ca_ inhibition by NS8593 was observed. The investigated dose of NS8593 increased the AF cycle length but was not sufficient to induce cardioversion. In terms of propagation properties during AF, *I*_K,Ca_ inhibition by NS8593 led to divergent effects in the right and left atrium. This divergent behavior may have impeded the cardioversion success.

## Introduction

Atrial fibrillation (AF) is the most common sustained cardiac arrhythmia, which poses a serious public health issue in Western societies as its prevalence increases drastically with age (Heeringa et al., 2006). It is estimated that one in four adults over the age of 40 in Europe and the United States will develop AF during the remainder of their lifetime, which is associated with a twofold increase in all-cause mortality and a markedly impaired quality of life (Kirchhof et al., 2016, 2020). The recently published Early Treatment of Atrial Fibrillation for Stroke Prevention Trial (EAST-AFNET 4) highlights the association between early rhythm-control therapy and a considerably lower risk of adverse cardiovascular outcomes (Kirchhof et al., 2020). Hence, the current unmet need for effective and safe pharmacological treatment options (Waks and Zimetbaum, 2017) vindicates further research efforts in the development of novel pharmacological treatment strategies (Milnes et al., 2012; El-Haou et al., 2015; Ravens, 2017).

Small-conductance Ca^2+^-activated K^+^ (K_Ca_2) channels have recently been proposed as a possible atrial-selective target to pharmacologically terminate AF (Diness et al., 2010). K_Ca_2 channel inhibition has indeed been shown to affect atrial repolarization in healthy human atrial myocytes and increase the atrial effective refractory period (aERP) in dissected atrial tissue strips (Skibsbye et al., 2014). However, the role of K_Ca_2 channels in persistent and permanent AF pathophysiology is still unclear.

A genome-wide association study revealed a possible relationship between gene variants encoding the K_Ca_2.3 channel and AF without detectable cause (Ellinor et al., 2010, 2012). Moreover, the atrial *I*_K,Ca_ current was shown to be enhanced after short-term atrial tachypacing in dogs (Qi et al., 2014). However, Skibsbye et al. (2014) reported a down-regulation of K_Ca_2.2 and K_Ca_2.3 mRNA in atrial cardiomyocytes from chronic AF patients, resulting in the absence of action potential duration (APD) and the aERP prolonging effect of K_Ca_2 inhibition. This was further supported in a canine model of heart failure combined with AF, where *I*_*K,Ca*_ inhibition did not prolong atrial APD (Bonilla et al., 2014). It may therefore be hypothesized that the importance of the K_Ca_2 current, and thereby the efficacy of *I*_K,Ca_ inhibition, depends on AF duration and the extent of AF-induced remodeling.

In contrast, various animal models for short- and longer-term AF reported aERP prolongation and decreased AF inducibility and stability in response to *I*_K,Ca_ inhibition, none of which had a considerable effect on ventricular electrophysiology (Diness et al., 2011, 2017; Skibsbye et al., 2011; Qi et al., 2014). A recent study investigating the anti-arrhythmic properties of the K_Ca_2 channel modulator NS8593 in horses with acutely induced AF paroxysms supported these findings (Haugaard et al., 2015). Allosteric K_Ca_2 channel modulation by NS8593 exerts an atrial-selective anti-arrhythmic class III drug effect and mechanistically decreases the Ca^2+^ sensitivity of K_Ca_2 channels, resulting in a decreased potassium outward current (Xu et al., 2003; Strøbæk et al., 2006; Sørensen et al., 2008). However, the prominent class III effect is accompanied by an indirect influence on Na^+^ channel availability (class I drug effect) (Skibsbye et al., 2015).

To address the hypothesis that the efficacy of *I*_K,Ca_ inhibition depends on AF duration and thereby the level of atrial remodeling, this high-density contact mapping study was designed to explore the anti-arrhythmic properties of NS8593 and its influence on both global and local atrial conduction during persistent AF.

In continuation of our previous work (Haugaard et al., 2015) we investigated NS8593 in a model of persistent AF in the horse. The central aim was to challenge AF stability by NS8593 and elucidate its electrophysiological effects on basic conduction properties, such as conduction velocity, complexity, and AF cycle length. These effects on basic conduction properties were measured on the left and right atrial free wall simultaneously to identify local and possible chamber specific effects.

## Materials and Methods

### Animals

A total of 11 Standardbred mares (461 ± 56 kg, height 158 ± 4 cm) with a mean age of 7 ± 3 years (range 3–12) were included in the study. The horses had no history of cardiovascular disease prior to the study, as confirmed by clinical examination, cardiac auscultation, 24-h Holter ECG monitoring, and echocardiographic examination. The specific inclusion protocol has been described previously (Carstensen et al., 2017).

**FIGURE 1:**
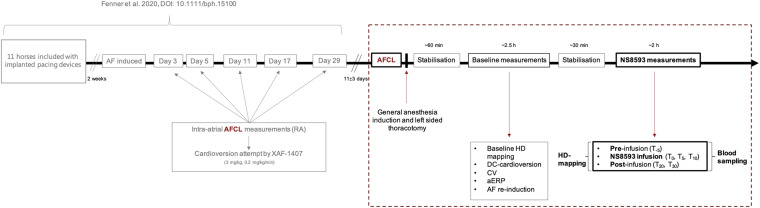
Experimental set-up and timeline in relation to the preceding study (Fenner et al., 2020). The preceding study was conducted using an equine model of tachypacing-induced persistent AF to investigate the effect of selective I_KACh_ inhibition by XAF-1407. The study was based on serial attempts of pharmacologically cardioverting AF over the course of 1 month. The present study (dashed box), investigating the inhibition of small-conductance calcium-activated potassium current (I_K,Ca_) in persistent AF in horses, succeeds Fenner et al. (2020) as illustrated above. The time-gap between both studies accounts for 11 ± 3 days, to ensure complete drug wash-out. The data acquired from the above illustrated experimental set-up, which is presented in this study, is marked in bold. Time points referred to throughout the main text and in Figures 2–7 are defined above (T_–5 to 30_). Source and acquisition time points of AFCL data presented in Figure 2 are marked in red. AF, atrial fibrillation; AFCL, atrial fibrillation cycle length; RA: right atrium.

The same horses were also included in a previous study, as this study is part of a series on the mechanisms underlying persistent AF and novel atrial-selective, target-based treatment strategies (Fenner et al., 2020). When enrolled in the present study, all horses had been under the influence of tachypacing-induced persistent AF for 42 ± 5 days (Figure 1).

While 11 horses entered the course of this study, five horses had to be excluded from mapping data analysis due to deficiencies in experimental conduct, which potentially influenced cardiovascular stability and thereby cardiac electrophysiology.

All experiments were performed at the Large Animal Teaching Hospital, Department of Veterinary Clinical Sciences, University of Copenhagen, Taastrup, Denmark.

The study was approved by the local ethical committee at the Department of Veterinary Clinical Sciences, University of Copenhagen and by the Danish Animal Experiments Inspectorate (license number 2016-15-0201-01128), and was performed in accordance with the European Commission Directive 86/609/ECC.

### AF Induction

A subcutaneous implantable cardioverter defibrillator in pacing mode (ICD; Maximo^®^ II, Concerto^®^; Medtronic Inc., Minneapolis, MN, United States; equipped with a specialized high rate pacing algorithm) and two right atrial bipolar pacing leads (Tendril^TM^ STS Pacing Leads, 100 cm, St. Jude Medical Inc., St. Paul, MN, United States) were implanted in all horses. Following implantation, all horses were treated with antibiotics (Benzylpenicillin^®^ Panpharma 3 g (5 mill./milj.IE/IU), Benzylpenicillin natr., Panpharma, Luitré, France), and non-steroidal anti-inflammatory drugs (Finadyne^®^ Vet. 50 mg/ml, Flunixin, IV, MSD, Intervet International B.V. AN Boxmeer, Netherlands) for a minimum of 3 days and were allowed a recovery period of 14 days. Subsequently, AF was induced by high-rate pacing (10 Hz) for ≥48 h or until AF was self-sustained. Longitudinal assessment of AF stability and progression was achieved by manual AF cycle length (AFCL) measurements from intra-atrial electrograms (Supplementary Figure 1) on days 3, 5, 11, 17, 29 (Fenner et al., 2020), and ∼40 after AF induction. The AF induction and maintenance protocol was part of a preceding study investigating the efficacy of pharmacological *I*_K,ACh_ inhibition in cardioverting AF of varying duration by serial cardioversion over a period of 1 month (Fenner et al., 2020).

### Open-Chest Mapping Procedure

The electrophysiological effects of K_Ca_2 current inhibition by NS8593 were evaluated in an open-chest experiment. The horses were intravenously premedicated with flunixin-meglumine (Finadyne^®^vet., MSD, Segré, France, 1.1 mg/kg), detomidine (Domosedan^®^vet., Orion Pharma Animal Health, Copenhagen, Denmark, 0.01 mg/kg), acetylpromazine (Plegicil^®^vet., Dechra Veterinary Products A/S, Uldum, Denmark, 0.03 mg/kg), morphine (Morfin DAK 20 mg/ml, Takeda Pharma A/S, Taastrup, Denmark, 0.06 mg/kg), and butorphanol (Torbugesic^®^, Orion Pharma Animal Health, Copenhagen, Denmark, 0.01 mg/kg). General anesthesia was induced by zolazepam combined with tiletamine (Zoletil^®^, Virbac Denmark A/S, Kolding, Denmark, 1.5 mg/kg i.v.) and maintained by isoflurane (IsoFlo Vet., Orion Pharma Animal Health, Copenhagen, Denmark, 1.4%). A constant-rate infusion of rocuronium (Rocuronium, Hameln Pharmaceuticals, Hameln, Germany, 0.3 mg/kg/h) was used for muscle relaxation. ECG and blood pressure were continuously monitored. Aortic pressure (P_Ao_) was measured using a pressure catheter (Pressure sensor, Sentron Europe BV, Leek, Netherlands) positioned in the ascending aorta *via* carotid artery access. A left-sided thoracotomy enabled simultaneous recording of atrial electrical activity by high-density contact mapping (249 electrodes, 2.5 mm inter-electrode distance; Supplementary Figure 2) on the epicardial surface of both atria (Adler et al., 2020). Unipolar AF electrograms were recorded at a sampling rate of 1,039 Hz. The signals were hardware filtered (1^st^ order high pass filter at 0.56 Hz, followed by a 1^st^ order low pass filter at 408 Hz) before digitization using a 16-bit analog-to-digital converter. To avoid interference of ventricular far-field, doubtful waveforms were detected (using a ventricular electrogram) and removed using averaged beat QRST-template cancellation.

### Drug Administration and Measurement of the Electrophysiological Parameters

The negative K_Ca_2 channel modulator NS8593 (5 mg/kg i.v, Acesion Pharma ApS) was administered over a 10-min period (Haugaard et al., 2015). Plasma levels in venous blood samples were measured at the time points *T* = −5, 0, 5, 10, and 30 min (where *T* = 0 was the start of drug administration) and subsequently analyzed at Syngene International Ltd., Bangalore, India. Plasma protein binding of NS8593 was determined using a standard plasma protein binding assay with tolbutamide as internal standard and warfarin as a reference compound. The assay is based on rapid equilibrium dialysis and subsequent compound detection by quantitative mass spectrometry (additional information in the Supplementary Material).

AF high density maps and ECG parameters were analyzed at the time points *T* = −5, 10, and 20 min. Mapping files of 10–60 s (dependent on availability) were analyzed to quantify the effects of NS8593 on conduction properties during AF. Local activation times (AT) were identified in all electrograms using a probabilistic algorithm (Zeemering et al., 2012). Based on the work of Kléber and Rudy (2004), we considered conduction block to occur if a putative conduction velocity (CV) of <20 cm/s was measured. Given the interelectrode distance of 2.5 mm, a maximal AT difference of 12 ms in the horizontal/vertical direction and a difference of 17 ms in the oblique direction was deducible. Conduction velocity was determined by plane-fitting through all activation time-points of the direct neighboring electrodes given the limits of 12 or 17 ms. Moreover, ATs were used to reconstruct fibrillatory waves. Wave boundaries were defined as the edge of the electrode or areas of conduction block. Fibrillation waves were classified as “peripheral” if the earliest activation site was located at the periphery of the array, and as a breakthrough if it was within the mapped area and could not be explained by epicardial conduction. Waves and breakthroughs were normalized to the cycle length. Re-entrant activity was identified based on conduction paths. Conduction paths were determined as the shortest continuous trajectory between a starting and end point of a wave, presuming a CV ≥ 20 cm/s. If the trajectory had ≥1 self-intersection after ≥75% of the mean AFCL it was defined as a local re-entry. Isolated activations of <3 adjacent electrodes were considered to be prone to noise or occasional miss-assignment of an AT. These waves were therefore not included in the analysis.

The anisotropy of conduction direction was determined at each electrode and was based on the circular variance of conduction vectors (Maesen et al., 2013).

ECG analysis to determine the heart rate (HR), QRS, and rate-corrected QT interval (QT_c_) was conducted for 10 subsequent RR intervals. The QT_c_ was corrected for HR using a piecewise linear regression model tailored to horses (Pedersen et al., 2016).

### Statistical Analysis

All data are displayed as median with IQR. Analyses were performed using GraphPad Prism 8 software (GraphPad Prism, RRID:SCR_002798) with *P* ≤ 0.05 considered significant. Parameters were tested for normality using a Shapiro–Wilk test. Conditions with <4 observations were not included in the analysis. The statistical method used was 2-way repeated measures ANOVA with time point as fixed and horse ID as a random variable to account for correlations over time. In this repeated-measures design, multiple comparisons were performed using Sidak’s test for multiple comparisons. The *post hoc* test was conducted only if the measure of matching effectivity achieved the necessary level of statistical significance (*P* ≤ 0.05), and significant variance inhomogeneity was not evident. A piecewise linear regression model has been used to correct QT intervals for HR on the data presented in Supplementary Figure 3.

## Results

### Atrial Fibrillation Maintenance and Atrial Remodeling

**FIGURE 2:**
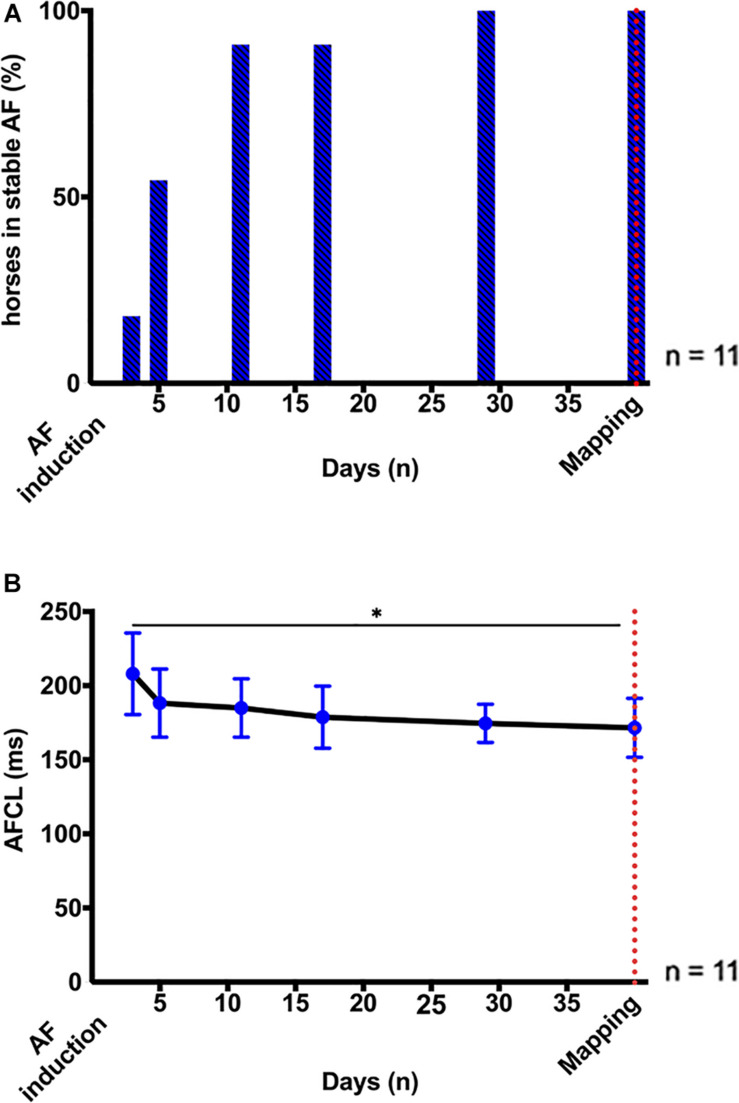
AF stabilization over 1 month. **(A)** Percentage (%) of horses in stable, self-sustained AF (≥ 24 h) after induction by high-rate atrial pacing (10 Hz). Induction of stable AF required 6 ± 4 days of pacing. **(B)** Longitudinal assessment of AF stability and progression by AF cycle length (AFCL) from right atrial intra-atrial electrogram recordings 3, 5, 11, 17, 29, and ∼40 days after AF induction. The red dotted line indicates the time point of the terminal high-density atrial mapping experiments. Statistical significance is defined as *p* < 0.05 and is marked with an asterisk (^∗^).

On average, AF became sustained after 6 ± 4 days of pacing at 10 Hz (Figure 2A). AF stabilization was accompanied by a progressive reduction in AFCL, from 209 (55) ms on day 3 to 169 (17) ms on day 29 (*p* < 0.05; Figure 2B), which indicated the progression of atrial electrical remodeling (Fenner et al., 2020).

### NS8593 Pharmacology and Pharmacokinetics

**FIGURE 3:**
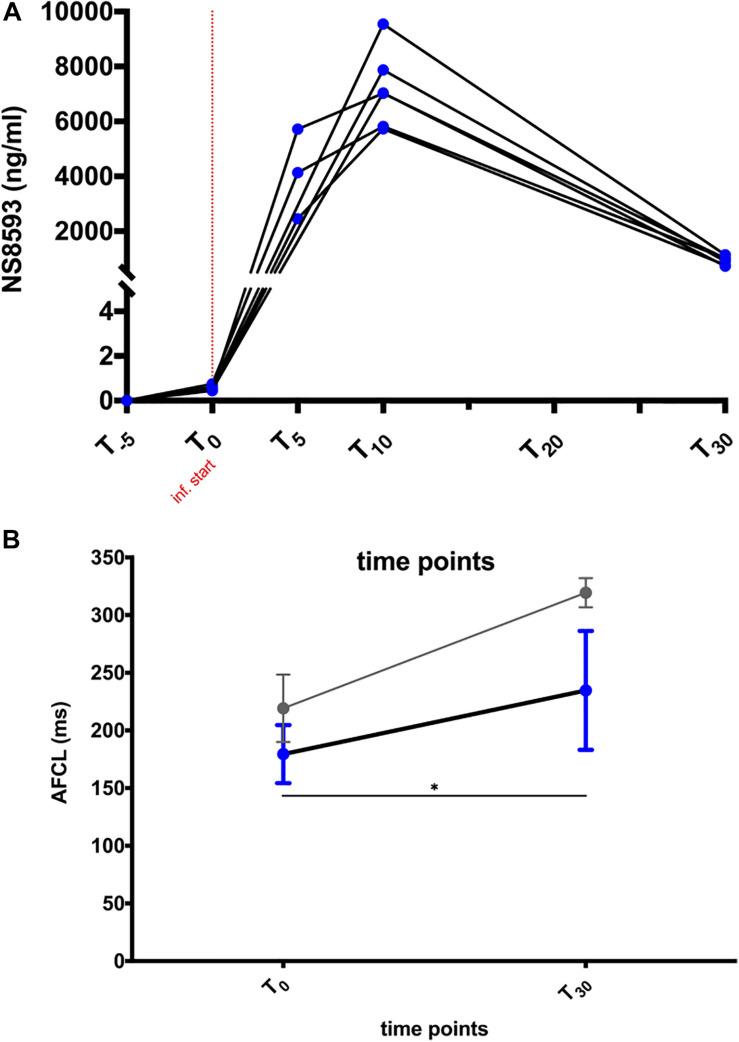
NS8593 pharmacokinetics and its effect on the atrial fibrillation cycle length (AFCL). **(A)** Plasma concentration of NS8593 measured at time points *T* = –5, 0, 5, 10, and 30 min relative to the start of drug infusion. **(B)** Influence of NS8593 on right atrial tissue refractoriness in persistent AF (blue) and acutely induced AF (gray). The depicted data on acute AF (gray) are derived from unpublished data from Haugaard et al. The shown data points represent the AF cycle length (AFCL) measured at the start of drug infusion (T_0_) and at the end of the 20-min observational period following the end of drug injection (T_30_, persistent AF study), as well as immediately before cardioversion (acute AF study), respectively. Inf., infusion. Statistical significance is defined as *p* < 0.05 and is marked with an asterisk (^∗^).

A maximal total plasma concentration (C_max_) of ∼7,200 ng/ml (27.4 μM) was reached at the end of infusion (Figure 3A). NS8593 had a plasma protein binding rate of 90.5% in equine plasma, corresponding to a free unbound plasma concentration of approximately 2.6 μM (9.5% of total plasma concentration) at C_max_. A rapid decrease in free plasma concentration to 0.33 μM (free unbound concentration) was observed over a period of 20 min after the end of infusion (Figure 3A). During those 20 min, the atrial fibrillatory process was monitored continuously using body surface ECG and atrial contact ECG recording to assess whether cardioversion to sinus rhythm occurred.

### Effect of NS8593 on Global and Local Conduction Properties During AF

NS8593 significantly prolonged AFCL by ∼50 ms [Figure 3B: RA: 185 (44) ms to 228 (81) ms; *p* < 0.05]. Interestingly, AFCL reached values similar to those measured 48 h after initial AF induction, when most animals had not yet developed sustained AF [See Figure 2B – day 3: 209 (55) ms and Figure 3B – T_30:_ 228 (81) ms)]. Despite this prolongation of AFCL, cardioversion of AF was not achieved.

**FIGURE 4:**
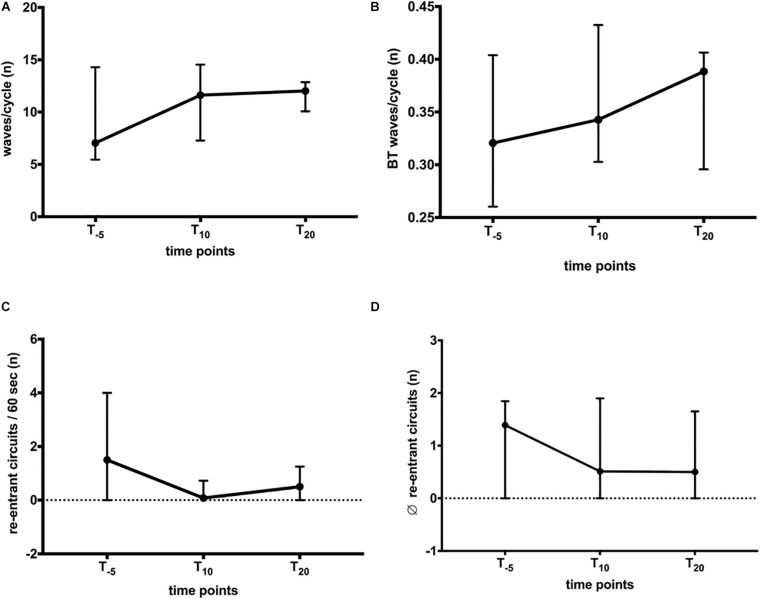
Global influence of NS8593 on conduction patterns contributing to AF maintenance. **(A)** No significant change in the total number of waves/cycle. **(B)** No significant change in the number of breakthrough waves relative to the number of waves/cycle. **(C)** No significant change in the total number of re-entrant circuits/second. **(D)** No significant change in the re-entrant circuit stability (average number of re-entrant circuit revolutions).

Wave patterns were determined to elucidate changes in conduction during AF. The recordings displayed various patterns, i.e., wavefront collision and fusion, breakthrough, and re-entrant circuits. These patterns were characterized by spatiotemporal instability, leading to a variety of patterns observed in horses and atria, respectively. The combined number of waves/AF cycle, breakthrough, and re-entries were quantified to capture this variety of patterns. We found no significant change in the total number of waves/AF cycle (LA + RA; Figure 4A) or in the relative number of breakthrough waves (Figure 4B). During a 60-s recording (Figure 4C), between 0 and 2 re-entries were observed, limited to 1.3 (4.7)% of the time (Figure 6C). The stability of observed re-entrant circuits was not affected by NS8593 (Figure 4D).

**FIGURE 5:**
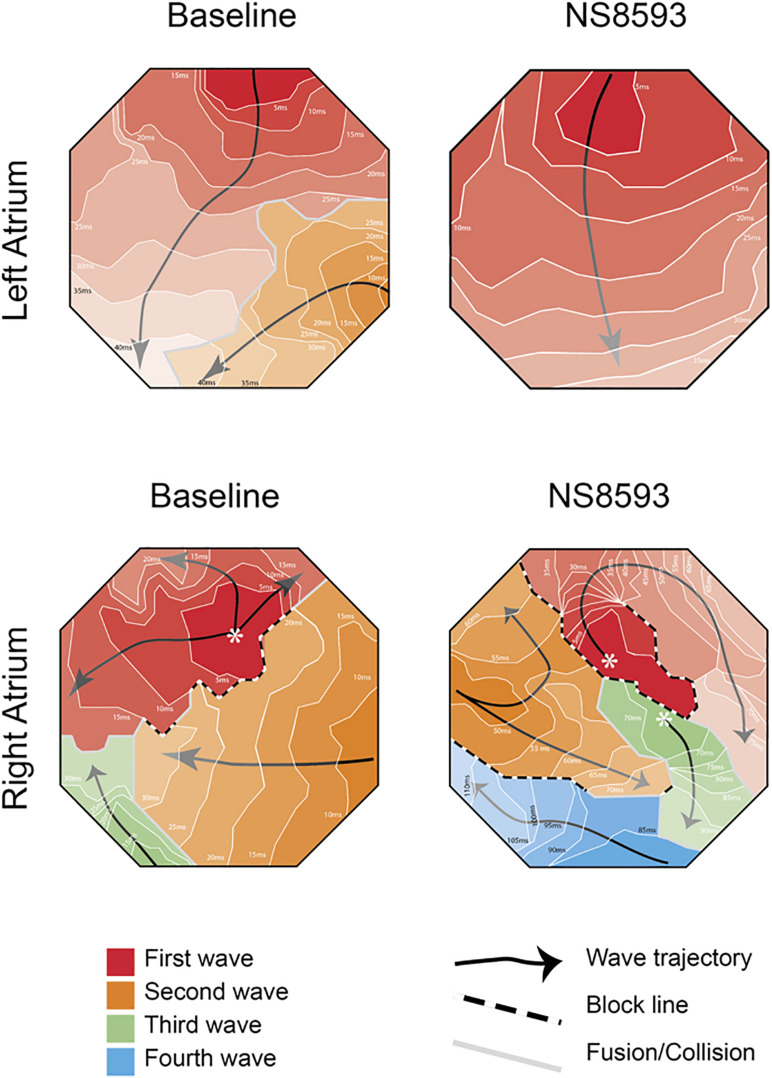
High-density bi-atrial contact mapping in the horse. Representative examples of isochronal maps recorded from right and left atrial free walls pre- and post-drug infusion. (Further information and isochronal maps can be found in Supplementary Material 4).

Representative activation maps from the right and left atrium are shown in Figure 5 before and after the administration of NS8593. Activation videos of 10 s of AF before and after drug administration are presented in the Supplementary Figure 4.

**FIGURE 6:**
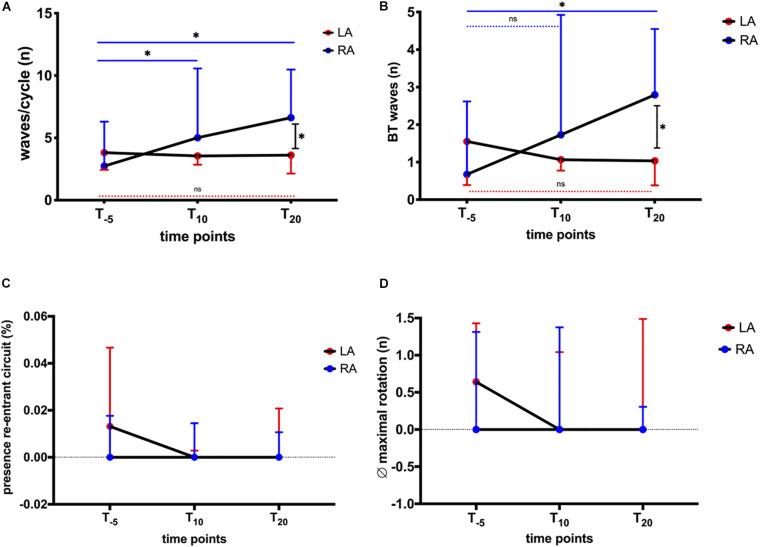
Influence of NS8593 on conduction patterns accountable for AF maintenance: ectopy and re-entrant circuits with focus on inter-atrial heterogeneity. AF parameters between baseline (T_–5_), 10 min (T_10_), and 20 min (T_20_) after the start of infusion in left atrium (LA) and right atrium (RA) **(A)** Waves/cycle. Significant increase in the number of waves/cycle in the RA, whereas no change can be observed for the LA. **(B)** Breakthrough waves. Significant increase in the number of breakthrough waves in the RA, whereas no change can be observed in the LA. **(C)** Presence of re-entrant circuits. As seen in Figure 3, re-entrant circuits were rarely observed events. There was no significant change in the percentage of time re-entrant circuits were present. **(D)** Average (∅) maximal rotation. Stability (maximal rotation) of observed re-entrant circuits was not significantly affected. Statistical significance is defined as *p* < 0.05 and is marked with an asterisk (^∗^).

When stratifying the changes in AF properties, we observed differences in behavior between the LA and RA. In the LA, the number of waves decreased after NS8593 infusion, while right atrial activation maps displayed narrower waves compared to the baseline. Prior to NS8593 infusion, the number of waves/cycle and breakthroughs displayed higher numbers in the LA [waves/cycle: 3.8 (7); breakthroughs: 1.6 (3.6)] compared to the RA [waves/cycle: 2.7 (4); breakthroughs: 0.7 (1.7); Figures 6A,B], however not statistically significant. There was no significant change in the number of waves and breakthroughs in the LA in response to NS8593 administration [waves/cycle: 3.6 (5); breakthroughs: 1 (1.6)]. In the RA, however, the number of waves and breakthroughs increased significantly [waves/cycle: 2.7 (4) to 6.6 (6), *p* < 0.05; breakthroughs: 0.7 (1.7) to 2.8 (3), *p* < 0.05], illustrating a distinct shift in the complexity gradient between the atria (*p* < 0.05).

**FIGURE 7:**
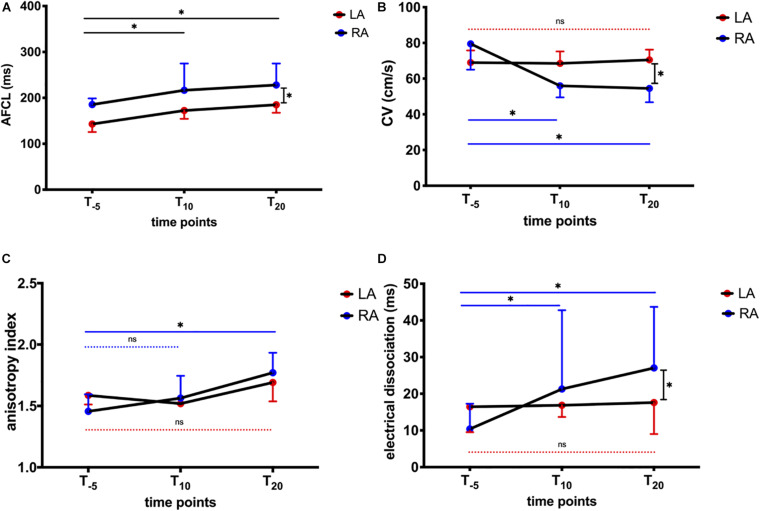
Influence of NS8593 on conduction properties. AF parameters between baseline (T_–5_), 10 min (T_10_), and 20 min (T_20_) after the start of infusion in left atrium (LA) and right atrium (RA) **(A)** AFCL. Left atrial AFCL is shorter than right atrial AFCL at T_0_. After NS8593 administration, both RA and LA AFCL increased equally, maintaining the RA-LA gradient. **(B)** CV_AF_. Significant decrease in conduction velocity in AF (CV_AF_) in the RA, whereas no change can be observed in the LA. **(C)** Anisotropy index. Significant increase in direction dependence (anisotropy) in the RA, whereas no change was observed in the LA. **(D)** Electrical dissociation. Significant increase in electrical dissociation in the RA, whereas no change was observed in the LA. Statistical significance is defined as *p* < 0.05 and is marked with an asterisk (^∗^).

We further dissected AFCL and conduction properties for RA and LA, respectively (Figure 7). LA AFCL was shorter compared to the RA (*p* < 0.05) at baseline. AFCL increased equally in the RA and LA following NS8593 administration, maintaining the same LA-RA gradient (Figure 7A). In contrast, NS8593 had a differential effect on the CV during AF (CV_AF_). Atrial CV_AF_ decreased significantly in the RA [from 79.5 (29) to 54.5 (17) cm/s, *p* < 0.05], while it remained unaffected in the LA [from 69 (13) to 70.5 (14) cm/s; Figure 7B]. The slowing of CV_AF_ in the RA was also associated with an increase in anisotropic conduction and dissociation (dyssynchrony) of activation times between neighboring electrodes (Figure 7C). The increase in electrical dissociation was particularly significant in the RA, whereas no change was observed in the LA (Figure 7D).

### Influence of NS8593 on Ventricular Electrophysiology and Hemodynamics

The RR interval, QRS duration, QTc interval, and mean P_*Ao*_ were assessed in 5-min intervals. A significant shortening of QTc (Supplementary Figure 3B, *p* < 0.05) was observed, associated with a trend toward shorter RR intervals (Supplementary Figure 3A). There was no significant change in the duration of the QRS complexes (Supplementary Figure 3C). In addition, no significant effects on systemic blood pressure or cardiovascular stability were noted (Supplementary Figure 3D).

## Discussion

To our knowledge, this is the first study to investigate the effect of antiarrhythmic drugs on conduction patterns in a horse model of sustained AF. Based on our previous work, we anticipated a strong possibility that *I*_K,Ca_ inhibition by NS8593 would terminate AF (Haugaard et al., 2015). However, restoration of sinus rhythm was not achieved in any of the investigated horses following NS8593 administration. The strong AFCL prolonging effect, beyond the point where AF previously had been unstable, suggests that a sufficient dose of NS8593 was administered to affect the electrical substrate in the horses, yet this effect was not accompanied by a reduction in AF complexity (number of waves). Interestingly, we were able to demonstrate differing effects of NS8593 on right- and left-atrial conduction properties. The effect on LA conduction was limited, while in the RA, conduction slowed down, anisotropy increased, and AF became more complex. It is conceivable that these effects on RA conduction impeded successful cardioversion.

### Influence of Pharmacological *I*_K,Ca_ Inhibition by NS8593 on Atrial Tissue Refractoriness

NS8593 has been shown to exert an *I*_K,Ca_ inhibitory effect by negative allosteric modulation of two specific amino acid residues located in the inner pore interacting with channel-specific gating structures (Jenkins et al., 2011). The resulting shift in Ca^2+^ sensitivity leads to a decrease in potassium outward current and thereby to APD and aERP prolongation (Simó-Vicens et al., 2017). Furthermore, K_Ca_2 channel inhibition has been suggested to reduce the fast sodium current: a mechanism that may explain the observed decrease in conduction velocity in the RA. Earlier it had been proposed that Na^+^ channel availability is indirectly influenced *via* the slight positive shift in the resting membrane potential; however, very recently, a direct inhibition of the sodium current by NS8593 in canine atria has been observed (Skibsbye et al., 2015; Burashnikov et al., 2020). This combination of these class I and III effects in NS8593 has previously been reported effective in horse with acute AF (Haugaard et al., 2015). However, in the present study, none of the horses cardioverted after 40 days of AF, even though a comparable unbound free fraction of NS8593 [2.6 μM (9.5%) at C_max_] was attained. The unbound free fraction of NS8593 was about ∼3 times higher than the IC_50_ for K_Ca_2.2 and K_Ca_2.3 in human atrial cells (Skibsbye et al., 2014), which may suggest that targeting K_Ca_2 channels alone is not sufficient in horses with persistent AF.

Despite the inability to terminate AF in these horses, NS8593 increased the AFCL substantially by ∼50 ms, corresponding to an AFCL prolongation, which effectively shortened and terminated AF paroxysms in acutely induced AF (Figure 3B). It can therefore be hypothesized that AF stability was further facilitated by additional remodeling processes. It is well known that the efficacy of currently available anti-arrhythmic drugs (AADs) to convert AF reduces with the progression of the atrial substrate (Eijsbouts et al., 2006; Kirchhof et al., 2016; Carstensen et al., 2018), which also seems to be the case for K_Ca_2 channel inhibition.

The apparent lack of cardioversion success despite the global AFCL prolongation of ∼50 ms to values similar to early AF progression agrees with previous observations in goats that the critical AFCL required for pharmacological cardioversion might increase substantially in longer-lasting AF (Eijsbouts et al., 2006). It could be hypothesized that further increasing the NS8593 concentration might have led to a sufficient increase in AFCL. However, this may result in a higher probability of non-specific ion channel block and subsequent loss of atrial specificity (Skibsbye et al., 2014).

When considering the pharmacological selectivity profile of NS8593 on relevant cardiac ion currents (Skibsbye et al., 2014), it is likely that some of the reported effects on atrial and ventricular electrophysiology can be attributed to not only the indirect but possibly also the direct class I drug effect (Burashnikov et al., 2020), as the free unbound C_max_ of 2.6 μM is comparable to the compound’s IC_50_ on Na_v_1.5 IC_50_ of 5 μM.

From the present study, we cannot conclude whether the lack of cardioversion was due to insufficient K_Ca_2 channel block, down-regulation of K_Ca_2 channels, or further substrate remodeling (including structural changes) to maintain persistent AF.

### AF Complexity and Inter-Atrial Heterogeneity

Similar to the apparent lack of any anti-arrhythmic effect of *I*_K,Ca_ inhibition in the present study, contrasting efficacy in pre-clinical drug testing has previously been reported for the class III AAD dofetilide. Dofetilide was highly effective in terminating “coarse” atrial fibrillatory patterns, whereas AF of higher complexity could not be terminated, even though AFCL was equally increased. This led to the assumption that class III drug efficacy might be significantly influenced by the underlying mechanism perpetuating AF (Li et al., 2000).

When investigating the coherence of increasing AF stability and the declining efficacy of currently available AADs, it has been shown that AF conduction patterns in the atrial free walls dissociate widely and thereby stabilize over the course of AF (Verheule et al., 2010). The presented differences in anti-arrhythmic efficacy of NS8593 in acute and persistent AF in horses therefore seem to be in agreement with the mechanistic findings of Verheule et al. as we also reported increased dissociated conduction in the right atrial free wall. The inter-atrial heterogeneity in AF complexity seen in response to NS8593 treatment in the persistent AF model is further supported by the class III AAD dofetilide influencing atrial electrophysiology toward stable and persisting AF patterns, particularly maintained by right atrial activity (Li et al., 2000). Similarly, NS8593 increased AFCL equally in both atria but did not affect complexity in the LA, while the RA complexity increased.

In accordance with several studies elaborating inter-atrial differences in AF propensity (Li et al., 2001; Verheule et al., 2010; Embi et al., 2014), LA activation maps reflected distinctively higher electrical complexity prior to drug infusion, suggesting that AF perpetuation was initially driven by left atrial electrical activity. In response to drug administration, however, CV decreased in the RA and remained unaffected in the LA, possibly preventing a global organization of the AF pattern. It, therefore, seems that the class III drug effect exerted by SK channel inhibition in the setting of persistent AF influences right atrial conduction in a way that supports AF perpetuation.

In a recent study investigating clinical AF cases characterized by left-to-right frequency gradients, it has been proven that elimination of the inter-atrial heterogeneity and AF complexity by ablation results in long-term SR maintenance (Atienza et al., 2009). This highlights the clinical importance of atrial specific investigation of pharmacological effects on cardiac electrophysiology in terms of conduction velocity and AF complexity in pre-clinical drug development as well as prospective clinical studies.

### Atrial Size and AF Perpetuation

Using the horse model of persistent AF raises the question of whether atrial size, and thereby substrate dimension, constitutes an important factor in AF stabilization and complexity (Kaese and Verheule, 2012). Comparisons made between mapping data in the goat and horse model of AF show a similar degree of AF complexity within the mapped area. Nevertheless, normalization of wave and path length to absolute atrial circumference suggests a relatively higher number of waves/cycle in the horse atria (Gatta et al., 2018). However, it seems unlikely that the evident lack of cardioversion by NS8593 treatment in this study is based on an initially higher relative AF complexity. On the contrary, the observed global increase in AFCL would give us reason to expect an increase in wave length (WL), effectively abrogating re-entrant circuits initially responsible for AF maintenance (Wang et al., 1993). However, as WL is the product of ERP and CV (WL = ERP × CV), the increase in global refractoriness seems to be equated by distinct uniatrial (RA) conduction slowing, ultimately preventing the anticipated increase in WL. Given the atrial size in horses, the lack of WL increase seems to allow for continuous activity, with exceptionally stable and more complex wavefronts observed in the RA contributing to AF perpetuation.

We further hypothesize that the observed local right atrial slowing of conduction, putatively due to indirect and possibly also direct sodium channel and/or gap junction blockade, facilitates the persistence of atrial fibrillatory activity. Right atrial activation maps display complex conduction patterns, further stabilized by increased anisotropy and lateral conduction failure in the mapped epicardial plane, known as “longitudinal dissociation” (Myerburg et al., 1973). Analogous conduction patterns of longitudinal dissociation, enhancing the AF complexity and stability, were likewise observed in a mapping study in long-term AF patients (Allessie et al., 2010). In these patients, lines of conduction block ran parallel to the right atrial pectinate muscles, offering a potential explanation for the RA-LA gradient in direction dependence, as left atrial trabeculae are oriented more randomly. It is conceivable that the same principle applies to the present study, as the anatomical structure of equine and human pectinate muscles has been reported to be comparable (Bright and Marr, 2010).

## Limitations

The present study contributes knowledge to experimental electrophysiology and provides novel insights into the *in vivo* pharmacology and electrophysiological properties of NS8593 inhibiting atrial *I*_K,Ca_ current. Using the horse as a new large animal model in cardiac electrophysiological research has both advantages and disadvantages. The size of the animal, and, as a consequence, the size of the atria, as well as the fact that horses are one of the few mammalian species besides humans that spontaneously develop AF mean that this species offers good translational value (Schüttler et al., 2020). However, due to the size of the species, studies are often limited to a relatively small number of animals. In the present study, a higher number of animals would have allowed us to set up a sham-operated control group to assess the effect of anesthesia and the open-chest setting on cardiovascular stability in order to differentiate between drug effect and unwanted interference with cardiac electrophysiology in the specific experimental setting. However, Haugaard et al. (2015) did not report any changes in aERP due to anesthesia.

Furthermore, a larger sample size might have allowed for species-specific dose-response experiments. However, given the well-known pharmacokinetic properties and the reported *in vitro* pharmacology, we would expect a significant portion of the *I*_K,Ca_ current to be inhibited.

Additionally, one technical limitation associated with atrial size must be considered – although both atria were mapped simultaneously, the mapped area was limited to the size of the electrode grid. As a result, it is possible that events might have taken place outside the field of view.

As mentioned in the result section, activation time videos illustrated that conduction patterns in AF are characterized by a high instability of spatiotemporal behavior (Supplementary Figure 4). This might affect the perception of the arrhythmia in short time samples, pilot analyses, however, revealed that the investigated parameters were not impacted by the reported minimal recording length of 10 s.

Lastly, future investigations to fully elucidate the effect of NS8593 and its derivatives in the presence of re-entrant circuits would be beneficial to further support our conclusions.

## Conclusion

A new open-chest *in vivo* model, including high-density contact mapping on the equine heart, has been developed. It allows detailed electrophysiological measurements and comparison between the LA and RA and was used to study the effect of K_Ca_2 channel inhibition by NS8593, exhibiting both class I and III anti-arrhythmic effects during AF.

In conclusion, our results have shown that selective inhibition of K_Ca_2 channels in horses with persistent AF leads to a global slowing of fibrillation frequency. However, the administered dose of NS8593, which successfully terminated acute AF, was not sufficient to lead to cardioversion in any of the included animals with persistent AF.

The observed differential effect on CV and AF complexity between the atria indicates inter-atrial differences in susceptibility for the indirect and direct class I drug effect of NS8593. In combination with the apparent coherence between an RA-LA frequency gradient in clinical AF and the capability of maintaining SR following cardioversion attempts (Atienza et al., 2009), this study’s findings highlight the importance of atrial specific investigation of pharmacological effects on cardiac electrophysiology in terms of basic conduction properties, such as conduction velocity, complexity and AF cycle length in pre-clinical drug development as well as prospective clinical studies.

Finally, as it seems like targeting K_Ca_2 channels alone is not sufficient to achieve a relevant prolongation of atrial tissue refractoriness in horses with persistent AF, experimental investigation of combinations of atrial-selective AADs may be considered in the future.

## Data Availability Statement

The raw data supporting the conclusions of this article will be made available by the authors, without undue reservation.

## Ethics Statement

The animal study was reviewed and approved by the Danish Animal Experiments Inspectorate (license number 2016-15-0201-01128).

## Author Contributions

MF, GG, AV, and RB contributed to the conception of the study. MF, GG, SS, TJ, SV, AV, and RB contributed to the design of the study. MF, GG, SS, MK, EH, DA, MS, SV, and AV contributed to the acquisition of data. MF, GG, SS, MK, EH, DA, MS, USc, USø, JD, TJ, SV, AV, and RB contributed to the intellectual content of the work by revising the draft until all authors gave their final approval for this version to be published. All authors agreed to be accountable for all aspects of the work and for ensuring that questions related to the accuracy or integrity of any part of the work are appropriately investigated and resolved.

## Conflict of Interest

USø and JD are co-founders of Acesion Pharma ApS and USø was one of the inventors of NS8593. The remaining authors declare that the research was conducted in the absence of any commercial or financial relationships that could be construed as a potential conflict of interest, as the anti-arrhythmic compound NS8593 was provided free of charge solely for academic purposes.

## Abbreviations

AAD: anti-arrhythmic drug
Aerp: atrial effective refractory period
AF: atrial fibrillation
AFCL: atrial fibrillation cycle length
APD: action potential duration
AT: activation time
BCL: basic cycle length
Ca^2+^: calcium
C_max_: maximal concentration
CV: conduction velocity
CV_AF_: conduction velocity during atrial fibrillation
HR: heart rate
ICD: implantable cardioverter defibrillator
*I*_K,Ca_: small-conductance Ca^2+^-activated K^+^ current
K^+^: potassium
K_Ca_2: small-conductance Ca^2+^-activated K^+^ channels
LA: left atrium
Na^+^: sodium
P_Ao_: aortic pressure
QTc: rate-corrected QT interval
RA: right atrium.

## References

[B1] Adler D. Hopster K. Hopster-Iversen C. Fenner M. Buhl R. Jacobsen S. (2020). Thoracotomy and pericardiotomy for access to the heart in horses: surgical procedure and effects on anaesthetic variables. *J. Equine Vet. Sci.* 2020:103315. 10.1016/j.jevs.2020.103315 33349415

[B2] Allessie M. A. De Groot N. M. S. Houben R. P. M. Schotten U. Boersma E. Smeets J. L. (2010). Electropathological substrate of long-standing persistent atrial fibrillation in patients with structural heart disease longitudinal dissociation. *Circulat. Arrhythmia and Electrophy.* 3 606–615. 10.1161/CIRCEP.109.910125 20719881

[B3] Atienza F. Almendral J. Jalife J. Zlochiver S. Ploutz-Snyder R. Torrecilla E. G. (2009). Real-time dominant frequency mapping and ablation of dominant frequency sites in atrial fibrillation with left-to-right frequency gradients predicts long-term maintenance of sinus rhythm. *Heart Rhythm* 6 33–40. 10.1016/j.hrthm.2008.10.024 19121797 PMC2867332

[B4] Bonilla I. M. Long V. P. Vargas-Pinto P. Wright P. Belevych A. Lou Q. (2014). Calcium-activated potassium current modulates ventricular repolarization in chronic heart failure. *PLoS One* 9:1–11. 10.1371/journal.pone.0108824 25271970 PMC4182742

[B5] Bright J. M. Marr C. M. (2010). *Introduction to cardiac anatomy and physiology. In Cardiology of the Horse* (Second Edi). Amsterdam: Elsevier Ltd, 10.1016/B978-0-7020-2817-5.00006-7

[B6] Burashnikov A. Barajas-Martinez H. Hu D. Robinson V. M. Grunnet M. Antzelevitch C. (2020). The Small Conductance Calcium-Activated Potassium Channel Inhibitors NS8593 and UCL1684 Prevent the Development of Atrial Fibrillation Through Atrial-Selective Inhibition of Sodium Channel Activity. *J. Cardiovasc. Pharmacol.* 76 164–172. 10.1097/FJC.0000000000000855 32453071 PMC7416459

[B7] Carstensen H. Hesselkilde E. Z. Fenner M. Loft-Andersen A. V. Flethøj M. Kanters J. K. (2018). Time-dependent antiarrhythmic effects of flecainide on induced atrial fibrillation in horses. *J. Vet. Int. Med.* 32:15287. 10.1111/jvim.15287 30133839 PMC6189357

[B8] Carstensen H. Kjær L. Haugaard M. M. Flethøj M. Hesselkilde E. Z. Kanters J. K. (2017). Antiarrhythmic Effects of Combining Dofetilide and Ranolazine in a Model of Acutely Induced Atrial Fibrillation in Horses. *J. Cardiovasc. Pharmacol.* 71:1. 10.1097/FJC.0000000000000541 29068807 PMC5768216

[B9] Diness J. G. Skibsbye L. Jespersen T. Bartels E. D. Sørensen U. S. Hansen R. S. (2011). Effects on atrial fibrillation in aged hypertensive rats by Ca2+-activated K+channel inhibition. *Hypertension* 57 1129–1135. 10.1161/HYPERTENSIONAHA.111.170613 21502564

[B10] Diness J. G. Skibsbye L. Simó-Vicens R. Santos J. L. Lundegaard P. Citerni C. (2017). Termination of Vernakalant-Resistant Atrial Fibrillation by Inhibition of Small-Conductance Ca2+-Activated K+ Channels in Pigs. *Circulat. Arrhythmia Electrophys.* 10 1–13. 10.1161/CIRCEP.117.005125 29018164 PMC5647113

[B11] Diness J. Sørensen U. S. Nissen J. D. Al-Shahib B. Jespersen T. Grunnet M. (2010). Inhibition of small-conductance ca2+-activated k+channels terminates and protects against atrial fibrillation. *Circulation* 3 380–390. 10.1161/CIRCEP.110.957407 20562443

[B12] Eijsbouts S. Ausma J. Blaauw Y. Schotten U. Duytschaever M. Allessie M. A. (2006). Serial cardioversion by class IC Drugs during 4 months of persistent atrial fibrillation in the goat. *J. Cardiovasc. Electrophys.* 17 648–654. 10.1111/j.1540-8167.2006.00407.x 16836716

[B13] El-Haou S. Ford J. W. Milnes J. T. (2015). Novel K+ channel targets in atrial fibrillation drug development - where are we? *J. Cardiovasc. Pharmacol.* 66 412–431. 10.1097/FJC.0000000000000277 25978691

[B14] Ellinor P. T. Lunetta K. L. Albert C. M. Glazer N. L. Ritchie M. D. Smith A. V. (2012). Meta-analysis identifies six new susceptibility loci for atrial fibrillation. *Nat. Genet.* 44 670–675. 10.1038/ng.2261 22544366 PMC3366038

[B15] Ellinor P. T. Lunetta K. L. Glazer N. L. Pfeufer A. Alonso A. Chung M. K. (2010). Common variants in KCNN3 are associated with lone atrial fibrillation. *Nat. Genet.* 42 240–244. 10.1038/ng.537 20173747 PMC2871387

[B16] Embi A. A. Scherlag B. J. Ritchey J. W. (2014). Glycogen and the propensity for atrial fibrillation: Intrinsic anatomic differences in glycogen in the left and right atria in the goat heart. *North Am. J. Med. Sci.* 6 510–515. 10.4103/1947-2714.143282 25489563 PMC4215488

[B17] Fenner M. F. Carstensen H. Nissen S. D. Hesselkilde E. Z. Lunddahl C. Jensen M. A. (2020). Effect of Selective I K, ACh Inhibition by XAF-1407 in an Equine Model of Tachypacing-induced Persistent Atrial Fibrillation (AF). *Br. J. Pharmacol.* 2020 1–17. 10.1111/bph.15100 PMC7393200 32436234

[B18] Gatta G. Fenner M. F. Sattler S. Schotten U. Jespersen T. Verheule S. (2018). Does size matter? Characterization of a horse model of chronic atrial fibrillation. *Heart Rhythm* 2018:B-PO04-019.

[B19] Haugaard M. Hesselkilde E. Pehrson S. Carstensen H. Flethøj M. Præstegaard K. (2015). Pharmacologic inhibition of small-conductance calcium-activated potassium (SK) channels by NS8593 reveals atrial antiarrhythmic potential in horses. *Heart Rhythm* 12 825–835. 10.1016/j.hrthm.2014.12.028 25542425

[B20] Heeringa J. Van Der Kuip D. A. M. Hofman A. Kors J. A. Van Herpen G. Stricker B. H. C. (2006). Prevalence, incidence and lifetime risk of atrial fibrillation: The Rotterdam study. *Eur. Heart J.* 27 949–953. 10.1093/eurheartj/ehi825 16527828

[B21] Jenkins D. P. Strobaek D. Hougaard C. Jensen M. L. Hummel R. Sorensen U. S. (2011). Negative Gating Modulation by (R)-N-(Benzimidazol-2-yl)-1,2,3,4-tetrahydro-1-naphthylamine (NS8593) Depends on Residues in the Inner Pore Vestibule: Pharmacological Evidence of Deep-Pore Gating of KCa2 Channels. *Mole. Pharmacol.* 79 899–909. 10.1124/mol.110.069807 21363929 PMC3102549

[B22] Kaese S. Verheule S. (2012). Cardiac electrophysiology in mice: A matter of size. *Front. Physiol.* 3:1–19. 10.3389/fphys.2012.00345 22973235 PMC3433738

[B23] Kirchhof P. Benussi S. Kotecha D. Ahlsson A. Atar D. Casadei B. (2016). 2016 ESC Guidelines for the management of atrial fibrillation developed in collaboration with EACTS. *Eur. Heart J.* 37 2893–2962. 10.1093/eurheartj/ehw210 27567408

[B24] Kirchhof P. Camm A. J. Goette A. Brandes A. Eckardt L. Elvan A. (2020). Early Rhythm-Control Therapy in Patients with Atrial Fibrillation. *N. Eng. J. Med.* 2020 1305–1316. 10.1056/nejmoa2019422 32865375

[B25] Kléber A. G. Rudy Y. (2004). Basic Mechanisms of Cardiac Impulse Propagation and Associated Arrhythmias. *Physiol. Rev.* 84 431–488. 10.1152/physrev.00025.2003 15044680

[B26] Li D. Bénardeau A. Nattel S. (2000). Contrasting efficacy of dofetilide in differing experimental models of atrial fibrillation. *Circulation* 102 104–112. 10.1161/01.CIR.102.1.104 10880422

[B27] Li D. Zhang L. Kneller J. Nattel S. (2001). Potential ionic mechanism for repolarization differences between canine right and left atrium. *Circulation Res.* 88 1168–1175. 10.1161/hh1101.091266 11397783

[B28] Maesen B. Zeemering S. Afonso C. Eckstein J. Burton R. A. B. Van Hunnik A. (2013). Rearrangement of atrial bundle architecture and consequent changes in anisotropy of conduction constitute the 3-dimensional substrate for atrial fibrillation. *Circulation* 6 967–975. 10.1161/CIRCEP.113.000050 23969531

[B29] Milnes J. T. Madge D. J. Ford J. W. (2012). New pharmacological approaches to atrial fibrillation. *Drug Disc. Today* 17 654–659. 10.1016/j.drudis.2012.02.007 22370250

[B30] Myerburg R. J. Nilsson K. Befeler B. Castellanos A. Gelband H. (1973). Transverse spread and longitudinal dissociation in the distal A-V conducting system. *J. Clin. Investig.* 1973:JCI107253. 10.1172/JCI107253 4693653 PMC302336

[B31] Pedersen P. J. Karlsson M. Flethøj M. Trachsel D. S. Kanters J. K. Klaerke D. A. (2016). Differences in the electrocardiographic QT interval of various breeds of athletic horses during rest and exercise. *J. Vet. Cardiol.* 18 255–264. 10.1016/j.jvc.2016.02.002 27068842

[B32] Qi X.-Y. Diness J. G. Brundel B. J. J. M. Zhou X.-B. Naud P. Wu C.-T. (2014). Role of small-conductance calcium-activated potassium channels in atrial electrophysiology and fibrillation in the dog. *Circulation* 129:430. 10.1161/CIRCULATIONAHA.113.003019 24190961

[B33] Ravens U. (2017). Atrial-selective K + channel blockers: potential antiarrhythmic drugs in atrial fibrillation? *Can. J. Physiol. Pharmacol.* 93 1313–1318. 10.1139/cjpp-2017-0024 28738160

[B34] Schüttler D. Bapat A. Kääb S. Lee K. Tomsits P. Clauss S. (2020). Animal Models of Atrial Fibrillation. *Circulation Res.* 127 91–110. 32716814 10.1161/CIRCRESAHA.120.316366

[B35] Simó-Vicens R. Kirchhoff J. E. Dolce B. Abildgaard L. Speerschneider T. Sørensen U. S. (2017). A new negative allosteric modulator, AP14145, for the study of small conductance calcium-activated potassium (KCa2) channels. *Br. J. Pharmacol.* 174 4396–4408. 10.1111/bph.14043 28925012 PMC5715977

[B36] Skibsbye L. Diness J. G. Sorensen U. S. Hansen R. S. Grunnet M. (2011). The duration of pacing-induced atrial fibrillation is reduced in vivo by inhibition of small conductance Ca(2+)-activated K(+) channels. *J. Cardiovasc. Pharmacol.* 57 672–681. 10.1097/FJC.0b013e318217943d 21394037

[B37] Skibsbye L. Poulet C. Diness J. G. Bentzen B. H. Yuan L. Kappert U. (2014). Small-conductance calcium-activated potassium (SK) channels contribute to action potential repolarization in human atria. *Cardiovascular Res.* 103 156–167. 10.1093/cvr/cvu121 24817686

[B38] Skibsbye L. Wang X. Axelsen L. N. Bomholtz S. H. Nielsen M. S. Grunnet M. (2015). Antiarrhythmic Mechanisms of SK Channel Inhibition in the Rat Atrium. *J. Cardiovasc. Pharmacol.* 66 165–176. 10.1097/FJC.0000000000000259 25856531

[B39] Sørensen U. S. Strøbæk D. Christophersen P. Hougaard C. Jensen M. L. Nielsen E. (2008). Synthesis and structure-activity relationship studies of 2-(N-substituted)-aminobenzimidazoles as potent negative gating modulators of small conductance Ca2+-activated K+ channels. *J. Med. Chem.* 2008:jm800809f. 10.1021/jm800809f 18998663

[B40] Strøbæk D. Hougaard C. Johansen T. H. Sørensen U. S. Nielsen E. Nielsen K. S. (2006). Inhibitory Gating Modulation of Small Conductance Ca ^2^^+^ - Activated K^+^ Channels by the Synthetic Compound (R) -N- (NS8593) Reduces Afterhyperpolarizing Current in Hippocampal CA1 Neurons. *Mole. Pharmacol.* 70 1771–1782. 10.1124/mol.106.027110.small 16926279

[B41] Verheule S. Tuyls E. Van Hunnik A. Kuiper M. Schotten U. Allessie M. (2010). Fibrillatory conduction in the atrial free walls of goats in persistent and permanent atrial fibrillation. *Circulation* 3 590–599. 10.1161/CIRCEP.109.931634 20937721

[B42] Waks J. W. Zimetbaum P. (2017). Antiarrhythmic Drug Therapy for Rhythm Control in Atrial Fibrillation. *J. Cardiovasc. Pharmacol. Ther.* 22 3–19. 10.1177/1074248416651722 27260643

[B43] Wang J. Bourne G. W. Wang Z. Villemaire C. Talajic M. Nattel S. (1993). Comparative mechanisms of antiarrhythmic drug action in experimental atrial fibrillation. Importance of use-dependent effects on refractoriness This article has been cited by other articles. *Circulation* 88 1030–1044. 8353865 10.1161/01.cir.88.3.1030

[B44] Xu Y. Tuteja D. Zhang Z. Xu D. Zhang Y. Rodriguez J. (2003). Molecular identification and functional roles of a Ca(2+)-activated K+ channel in human and mouse hearts. *J. Biol. Chem.* 278 49085–49094. 10.1074/jbc.M307508200 13679367

[B45] Zeemering S. Maesen B. Nijs J. Lau D. H. Granier M. Verheule S. (2012). Automated quantification of atrial fibrillation complexity by probabilistic electrogram analysis and fibrillation wave reconstruction. *Proc. Ann. Int. Con. IEEE Eng. Med. Biol. Soc.* 2012 6357–6360. 10.1109/EMBC.2012.6347448 23367383

