# Supplementary material for: Inhibition of Small-Conductance Calcium-Activated Potassium Current (IK,Ca) Leads to Differential Atrial Electrophysiological Effects in a Horse Model of Persistent Atrial Fibrillation
Source: Front Physiol. 2021 Feb 9;12:614483. doi: 10.3389/fphys.2021.614483 (PMC7900437; doi:10.3389/fphys.2021.614483)
Supplement: Supplementary file 1 [file Data_Sheet_1.PDF]

Supplementary Material

1 Supplementary Material and Methods

1.0 Pharmacokinetic analyses

The plasma protein binding of NS8593 in equine samples has been analyzed using a standardized human plasma protein binding assay with the following details. Warfarin was used as standard reference, as it is a well-known high bound compound.

Human Plasma protein binding assay

**Category**	**Syngene Protocol**
**Experimental Setup**	
Species	HORSE
Matrix Source	Provded by client
Buffer	Phosphate Buffered Saline pH 7.4
Compound concentration	10 µM
Method	Rapid Equilibrium Dialysis Method (RED)
Organic solvent during incubation	< 1%
Incubator	Thermomixer comfort
Replicate incubations per matrix	2
Incubation temperature	37°C
Sample time points	5 hr
Shaking	450 rpm
Aliquots	50 µl of plasma and 50 µl of Buffer after 5 hrs
**Sample Preparation**	
Reaction Termination	By adding 3 volumes of Acetonitrile containing Internal Standard
Internal Standard	Tolbutamide, 0.5µg/ml
**Sample processing**	
Vortex	5 min at 1200 rpm
centrifugation	10 min at 4000 rpm
**Sample Analysis**	
Detection Technique	LC-MS/MS
LC-MS/MS system	API 4500 coupled to Shimadzu Nexera-UHPLC
Quantitation	MRM
**Data Delivery**	
Report format	excel
peak area	analyte, Internal Standard
peak area ratio	analyte to internal standard at 5 hr
Deliverables	% Fraction unbound, % Bound & % Recovery

Formula

Percent fraction unbound = (Drug conc in buffer after 5hr/Drug conc in plasma after 5hr)*100

Percent fraction bound= 100 - Percent fraction unbound

% recovery=(100*(((area ratio of plasma)*(vol of plasma))+((area ratio of buffer)*(vol of buffer)))/(area ratio of T0)*(vol of T0))

Samples for total plasma concentration determination were prepared using a protein precipitation (PPT) extraction technique followed by liquid chromatography coupled to a tandem mass spectrometer. Further details are summarized below:

	Sample Preparation
Protein Precipitation - Method	Horse Plasma Calibration standards and Quality Control Standards were prepared by serial dilution
	From the above stocks 10 µL of Horse Plasma Standards, Quality Control Standards and Study samples were transferred to 1.1 ML 96 well Plate.
	To above samples 200 µL of Acetonitrile containg IS (500ng\ml-Tolbutamide) was added.
	All samples were vortexed for 5 min and centrifuged at 4000 rpm for 10 min.
	180 µL of supernatant was Transferred and injected into LC-MS/MS system

Chromatographic Conditions

Mass Spectra	API-4500 (LC-MS/MS) system with analyst 1.6.3
HPLC	Nexera X2 LC
Column	KINETIX C18, 50*2.1mm, 5µ
Mode	APCI Positive Mode

**Mobile Phase**	
Method	Binary gradient
Buffer	0.1% Formic Acid in Water
**Composition**	
Pump-A	0.1% Formic Acid in Water
Pump-B	0.1% Formic acid in Acetonitrile
Flow Rate	600 µL/min
Run time	3.0 Min
Injection Volume	2 µl
Column Oven	40 °C
Autosampler Temperature	8°C
Scan Mode	Positive mode

	Time (min)	% A	% B
**Gradient Condition**	0	98	2
	0,3	98	2
	2,1	2	98
	2,5	2	98
	2,6	98	2
	3	98	2

**MRM Conditions**	Name	Q1	Q3	Dwell time(msec)	DP	EP	CE	CXP
Compound Blood	NS-8593	264,1	134	50	50	10	22	10
IS	Tolbutamide	271,2	91,2	40	70	10	45	11

	Parameters	Values
**Source Parameters**	Curtain gas	45
	CAD Gas	10
	GS 1	45
	GS 2	45
	Nebulizer Current (NC)	5
	Temp	550
	Interface Heater	On

2 Supplementary Figure S1

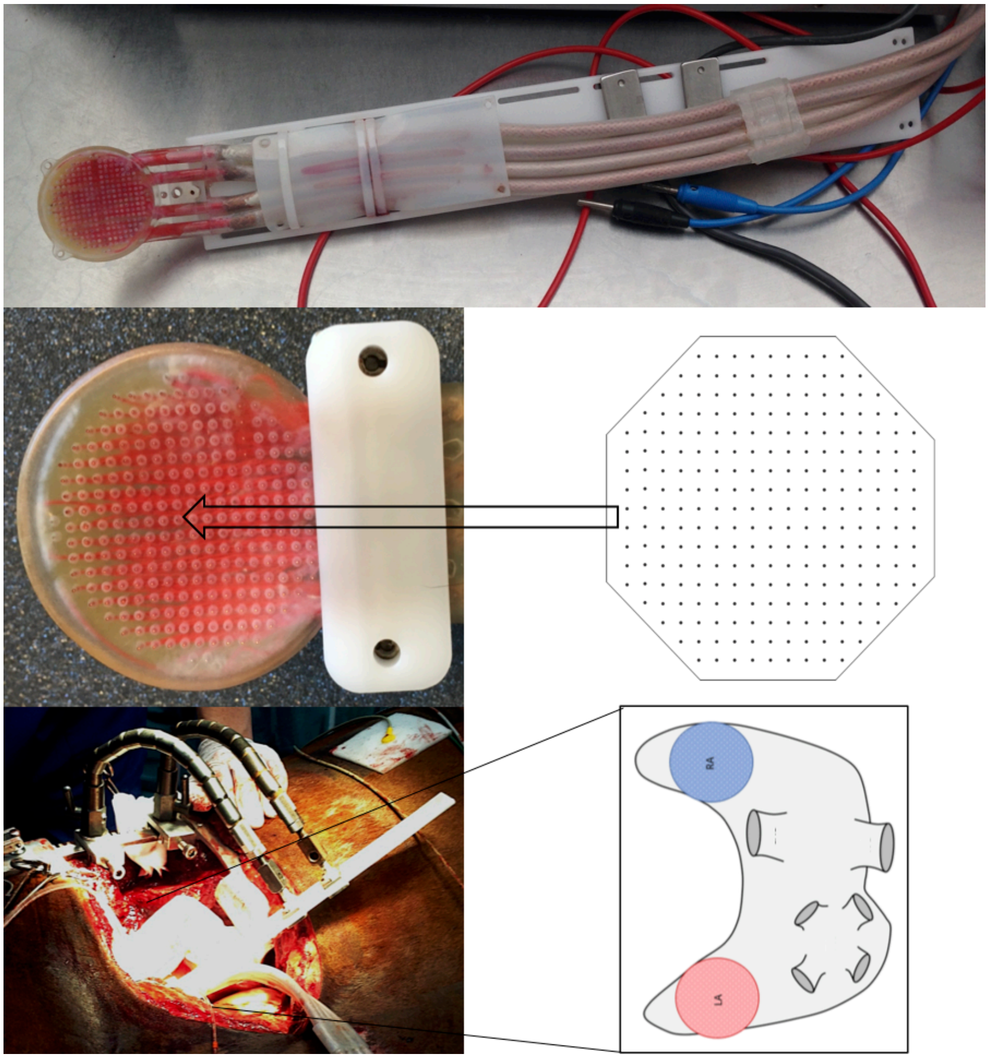

Figure S1. Technical data of the HD mapping tool and anatomical schematic of the mapping site.

The round, cup-shaped carrier of the mapping electrode is made of polycarbonate and can be mounted to variable reinforcing shafts suitable to the investigation site (top). An octagonal grid of silver electrodes (∅ 0.25mm) is employed within the carrier (middle). The mapping tool is kept in position by articulation arms, allowing for accurate adjustment of the mapping site (left bottom corner). The horses were placed in right-lateral recumbency, resulting in an approximate orientation of the mapping site as illustrated in the anatomical schematic drawing (right bottom corner). The carriers are covering approximately ¼ of the respective atrial free wall.

3 Supplementary Figure S2

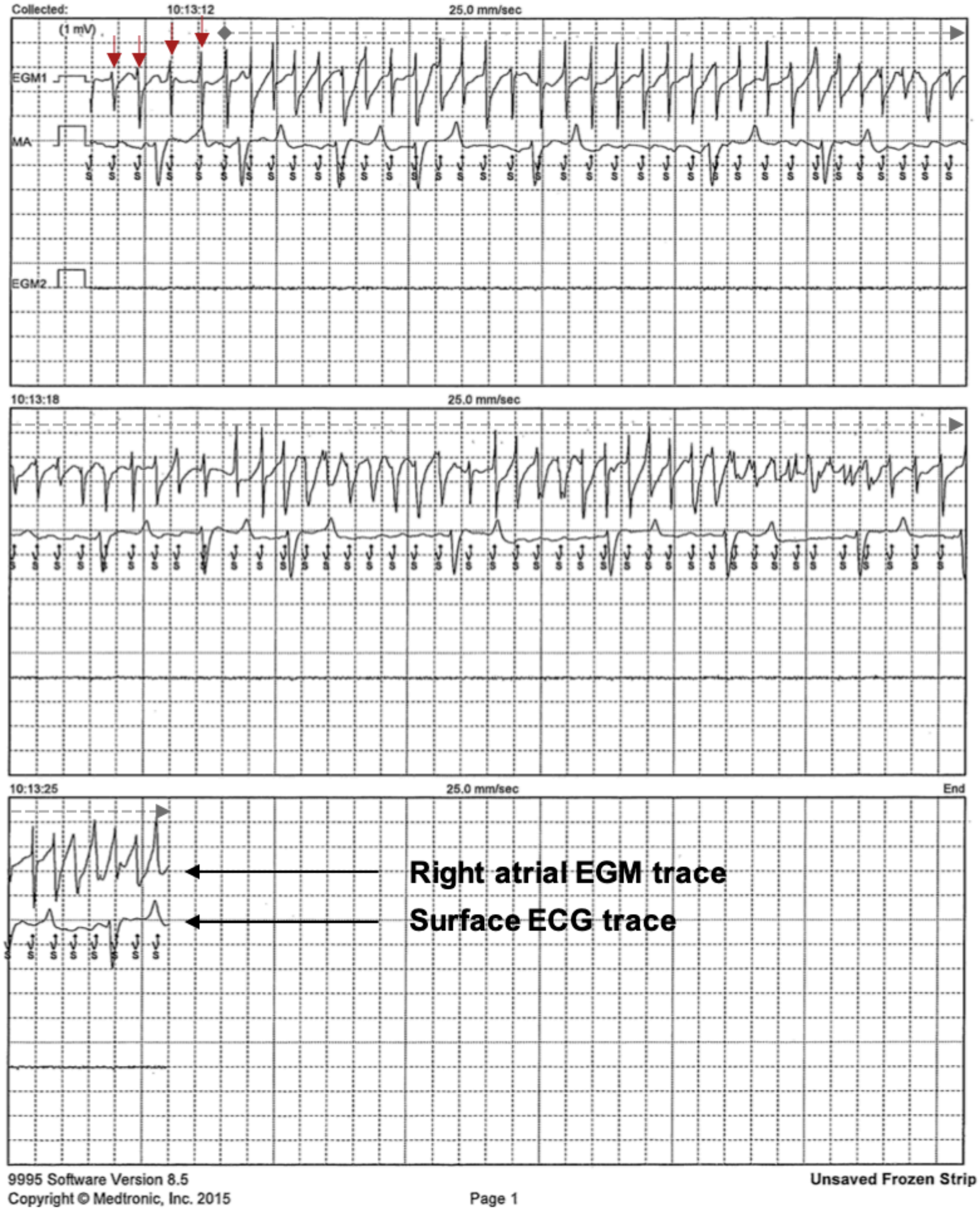

Figure S2. Exemplary intra atrial electrogram for AFCL calculation (incl. corresponding surface ECG trace)**.**

The intra atrial electrograms were collected from the right atrial free wall using bipolar pacing leads (Tendril™ STS Pacing Leads, 100 cm; St. Judes Medical Denmark A/S, Glostrup, Denmark) connected to dual- chamber ICDs. The readout was done through a Medtronic Carelink Programmer.

AFCL was analyzed manually by counting consecutive positive atrial deflections (peaks; red arrows) from EGM recordings, dividing the total recording time (14 s; grey arrow) through the peak count:

$$AFCL = \frac{14{,}000\ ms}{EGM\ peaks}$$

4 Supplementary Figure S3

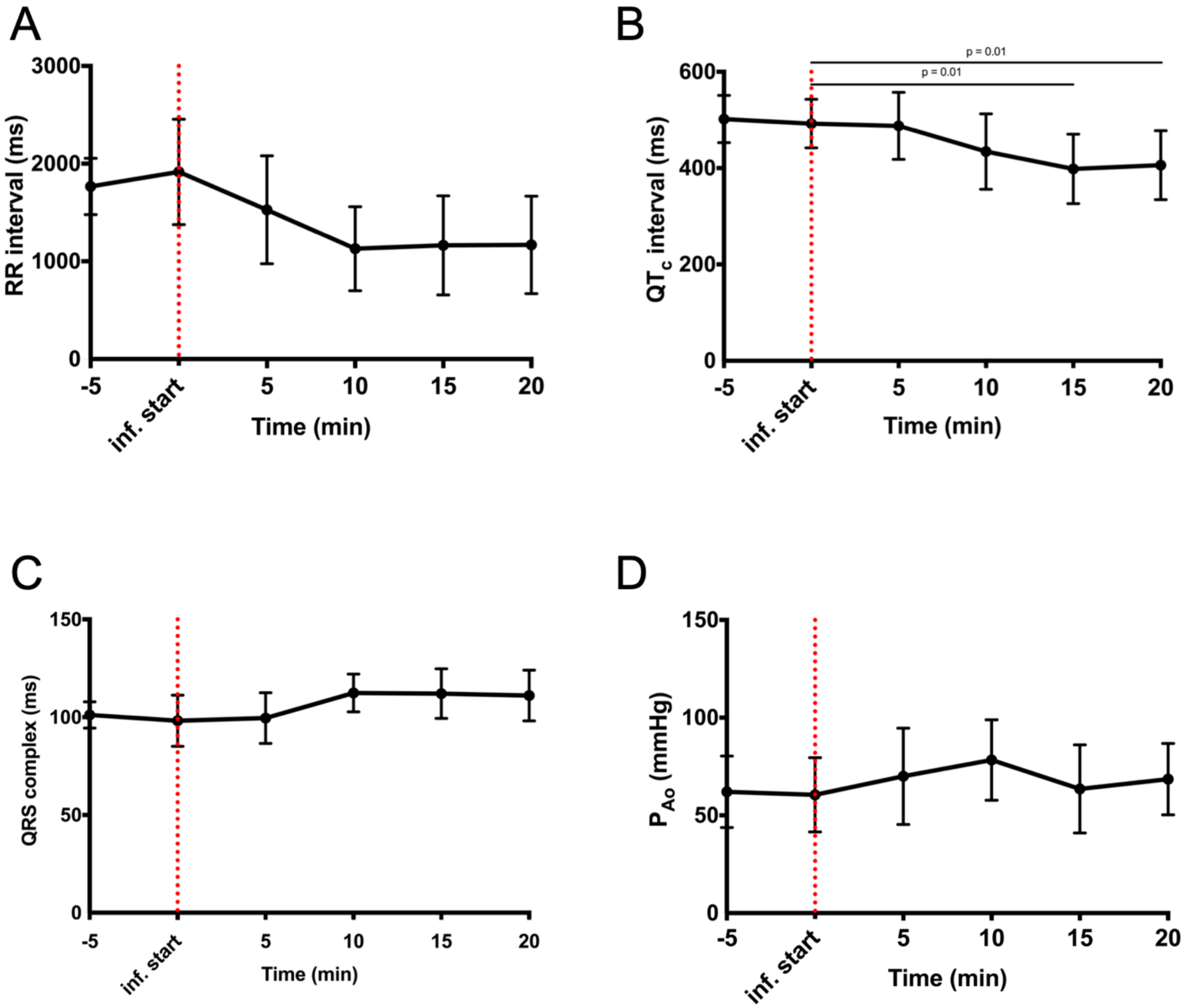

Figure S3. Effect of NS8593 on surface ECG parameters and hemodynamics.

(A) RR-intervals. During drug infusion the RR-intervals decreased (ventricular rate increased), however not significantly (p= 0.2). **(B) QTc–intervals**. In concurrence with the reported increase in HR, QTc- intervals expressed shortening of ventricular repolarisation. **(C) QRS – complex**. QRS-complexes became wider towards the end of injection, however non-significant (p= 0.2) and within the physiological range for horses (≤ 140 ms (Verheyen et al. 2010)). **(D) Aortic pressure (P_{Ao})**. No significant changes in P_{Ao} were observed (p = 0.5).

Statistical significant difference is defined as p< 0.05.

Verheyen, T., A. Decloedt, D. De Clercq, P. Deprez, S. U. Sys, and G. Van Loon. 2010. "Electrocardiography in Horses - Part 1: How to Make a Good Recording." *Vlaams Diergeneeskundig Tijdschrift* 79 (5): 331–36.
