# Supplementary material for: Inhibition of Small-Conductance Calcium-Activated Potassium Current (IK,Ca) Leads to Differential Atrial Electrophysiological Effects in a Horse Model of Persistent Atrial Fibrillation
Source: Front Physiol. 2021 Feb 9;12:614483. doi: 10.3389/fphys.2021.614483 (PMC7900437; doi:10.3389/fphys.2021.614483)
Supplement: Supplementary file 2 [file Presentation_1.PPTX]

## Slide 1
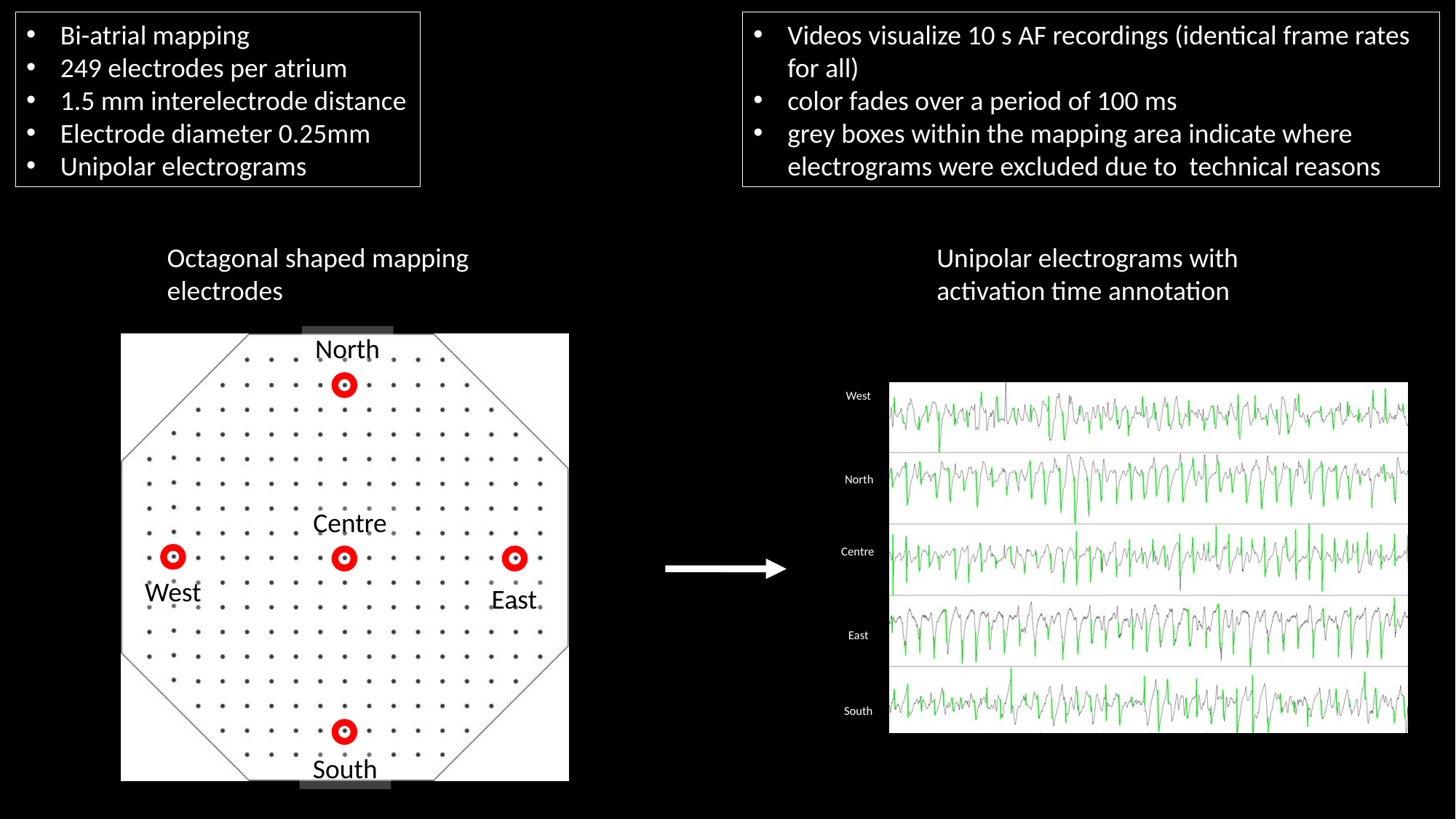

Bi-atrial mapping
249 electrodes per atrium
1.5 mm interelectrode distance
Electrode diameter 0.25mm
Unipolar electrograms
Videos visualize 10 s AF recordings (identical frame rates for all)
color fades over a period of 100 ms
grey boxes within the mapping area indicate where electrograms were excluded due to technical reasons
Octagonal shaped mapping electrodes
Unipolar electrograms with activation time annotation
North
Centre
West
East
South
West
North
Centre
East
South
