# Supplementary figures and images for: Inhibition of Small-Conductance Calcium-Activated Potassium Current (IK,Ca) Leads to Differential Atrial Electrophysiological Effects in a Horse Model of Persistent Atrial Fibrillation
Source: Front Physiol. 2021 Feb 9;12:614483. doi: 10.3389/fphys.2021.614483 (PMC7900437; doi:10.3389/fphys.2021.614483)

## Slide 1
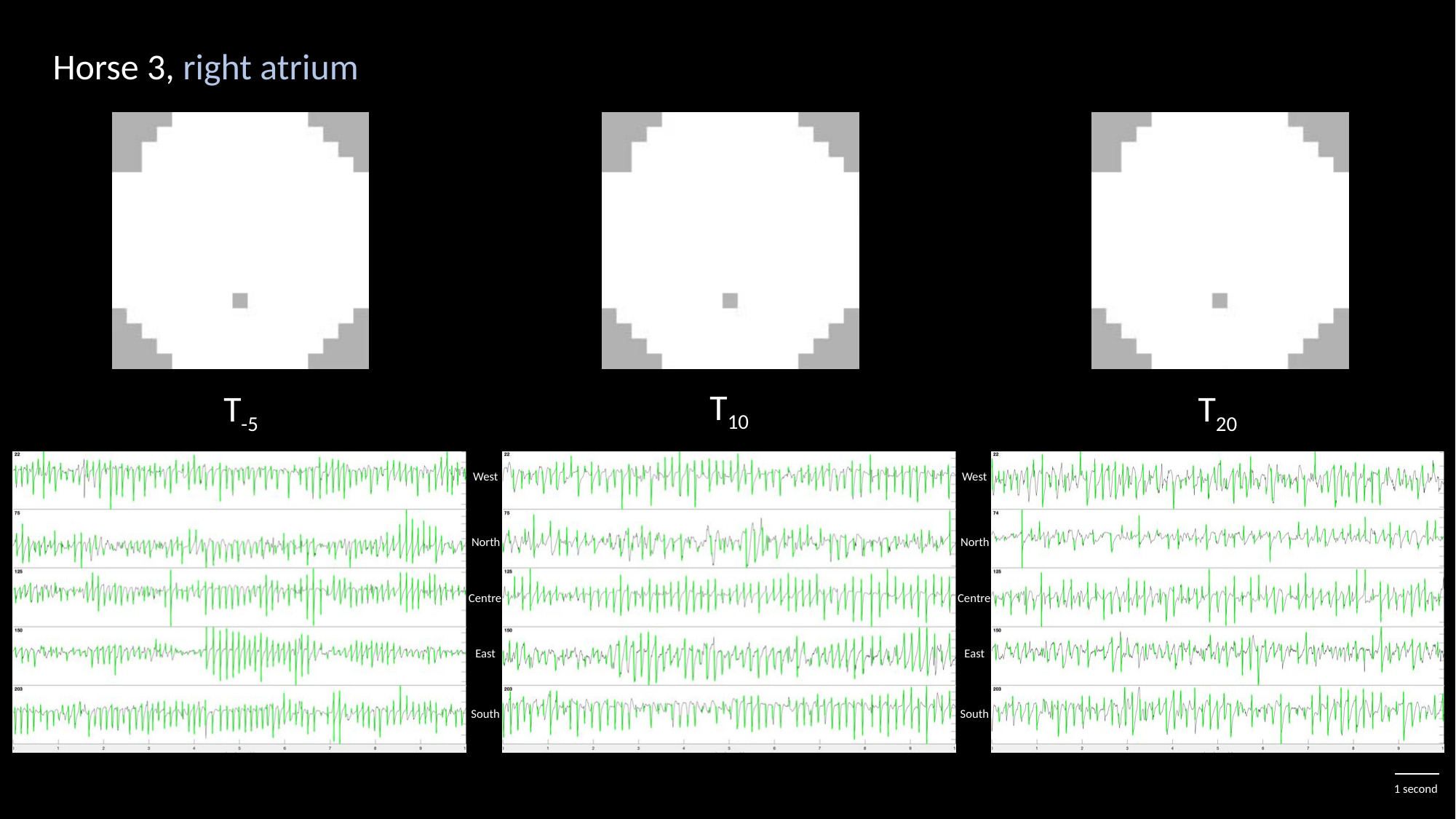

Horse 3, right atrium
T10
T20
T-5
West
North
Centre
East
South
West
North
Centre
East
South
1 second

Supplement: Supplementary file 3 [file Presentation_2.PPTX]

## Slide 1
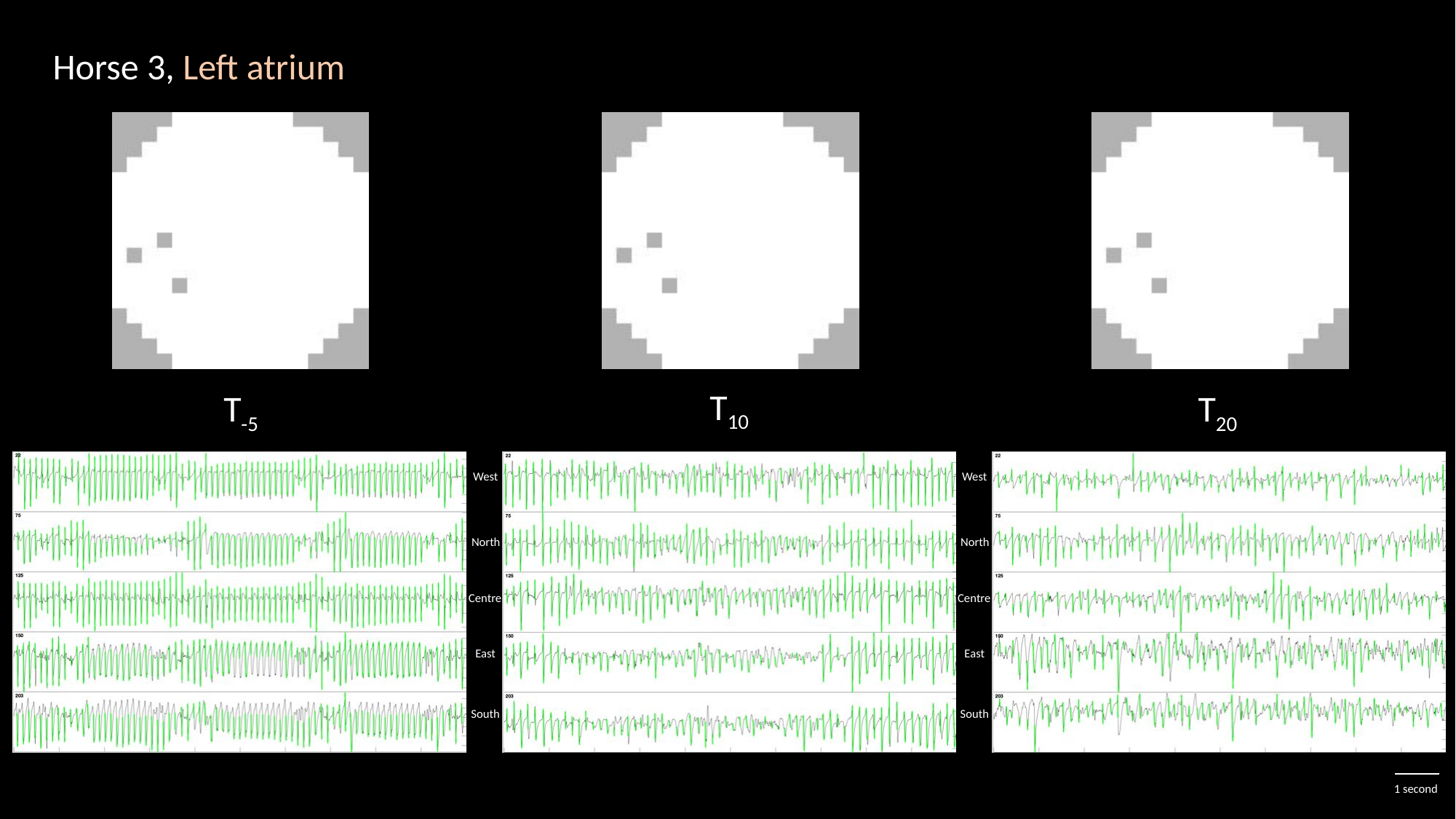

Horse 3, Left atrium
T10
T20
T-5
West
North
Centre
East
South
West
North
Centre
East
South
1 second

Supplement: Supplementary file 4 [file Presentation_3.PPTX]

## Slide 1
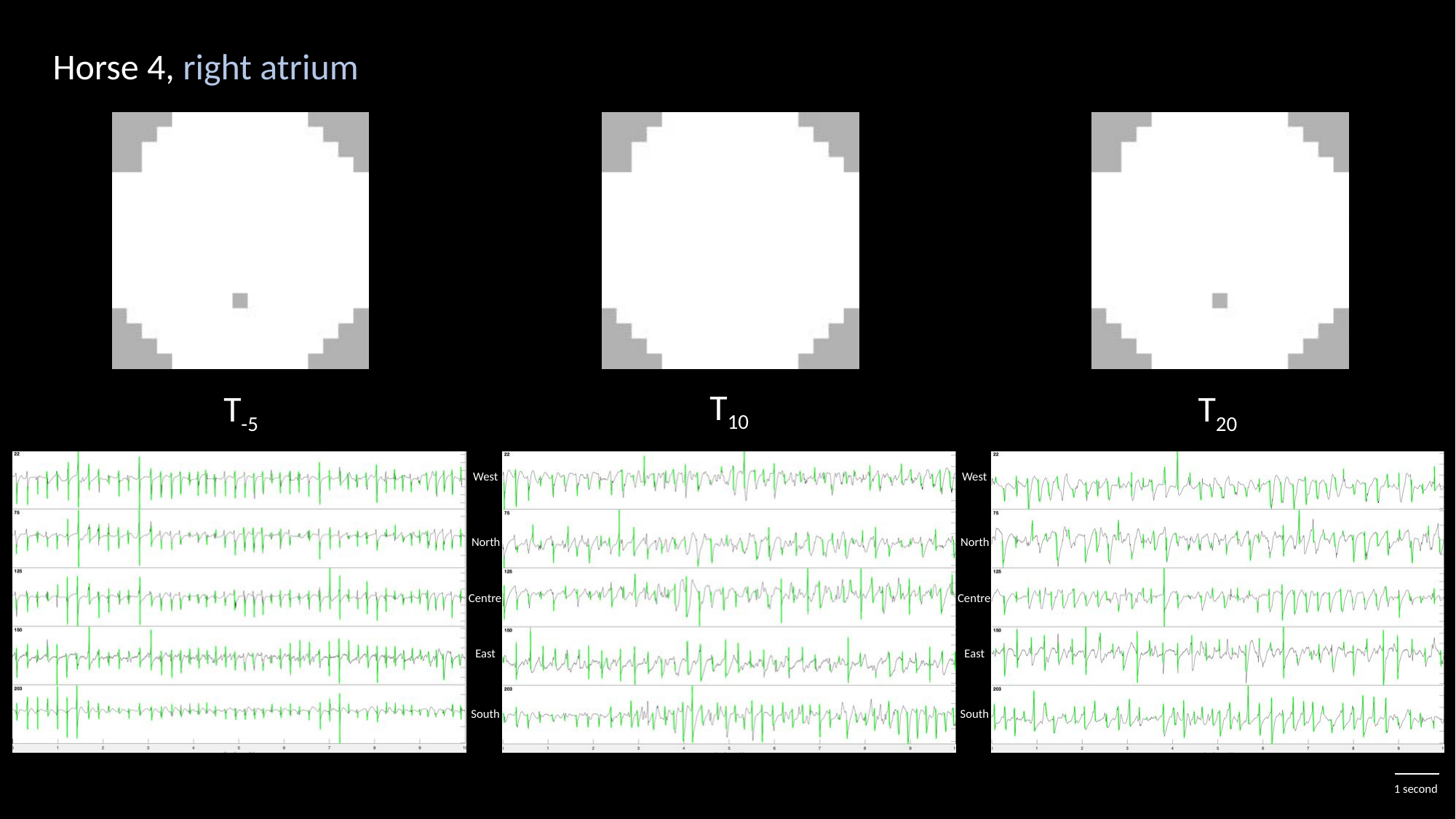

Horse 4, right atrium
T10
T20
T-5
West
North
Centre
East
South
West
North
Centre
East
South
1 second

Supplement: Supplementary file 5 [file Presentation_4.PPTX]

## Slide 1
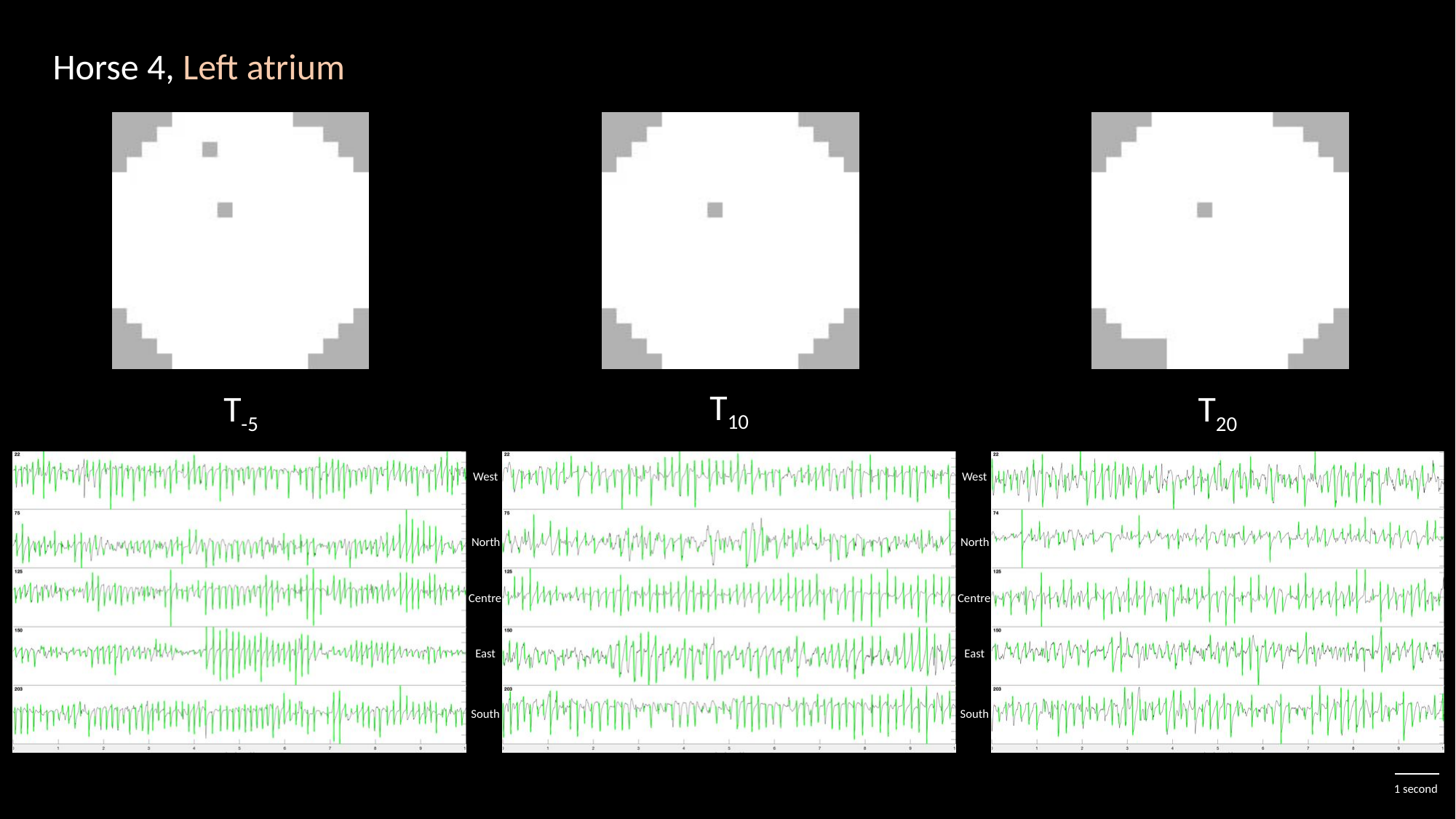

Horse 4, Left atrium
T10
T20
T-5
West
North
Centre
East
South
West
North
Centre
East
South
1 second

Supplement: Supplementary file 6 [file Presentation_5.PPTX]

## Slide 1
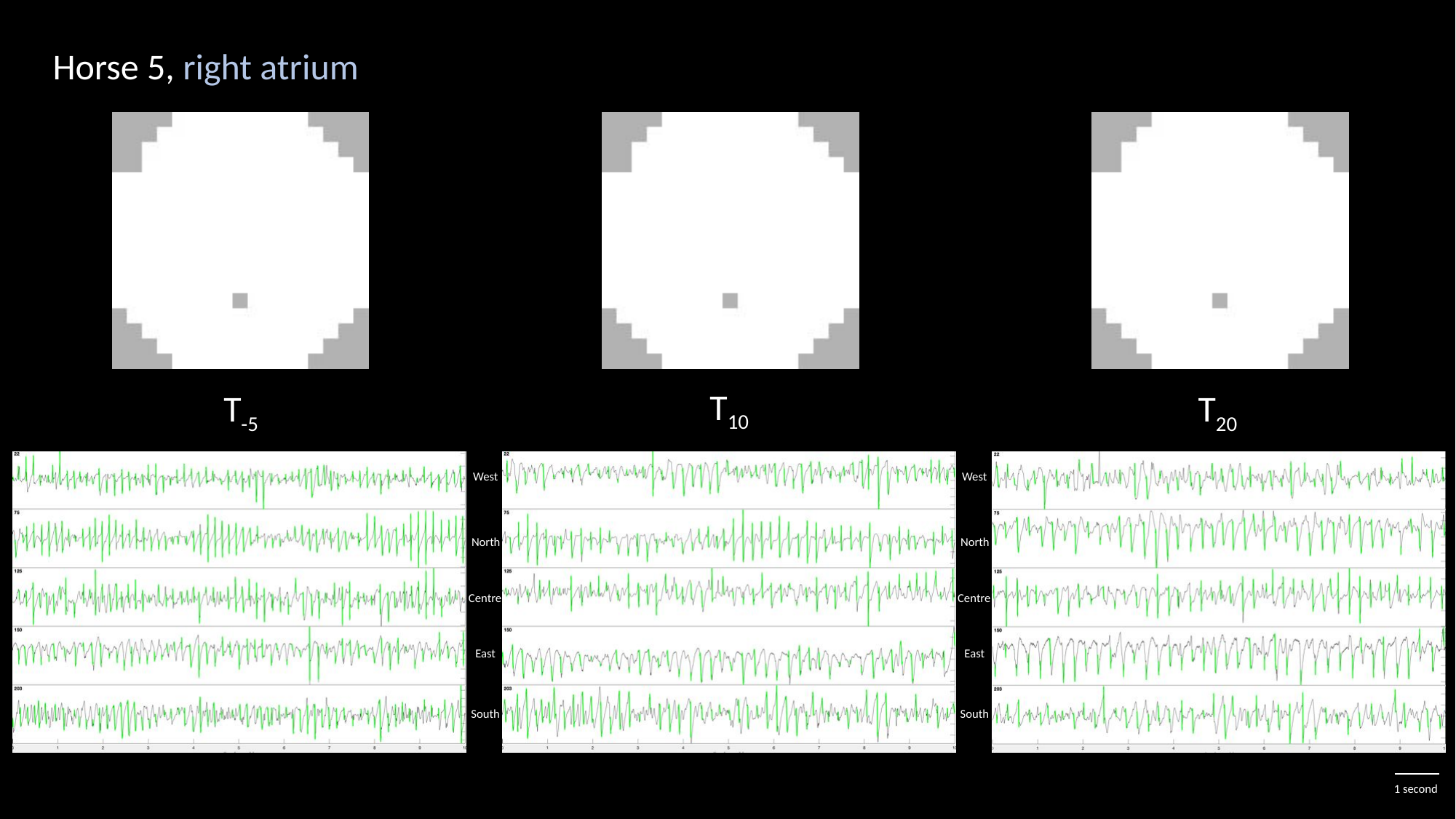

Horse 5, right atrium
T10
T20
T-5
West
North
Centre
East
South
West
North
Centre
East
South
1 second

Supplement: Supplementary file 7 [file Presentation_6.PPTX]

## Slide 1
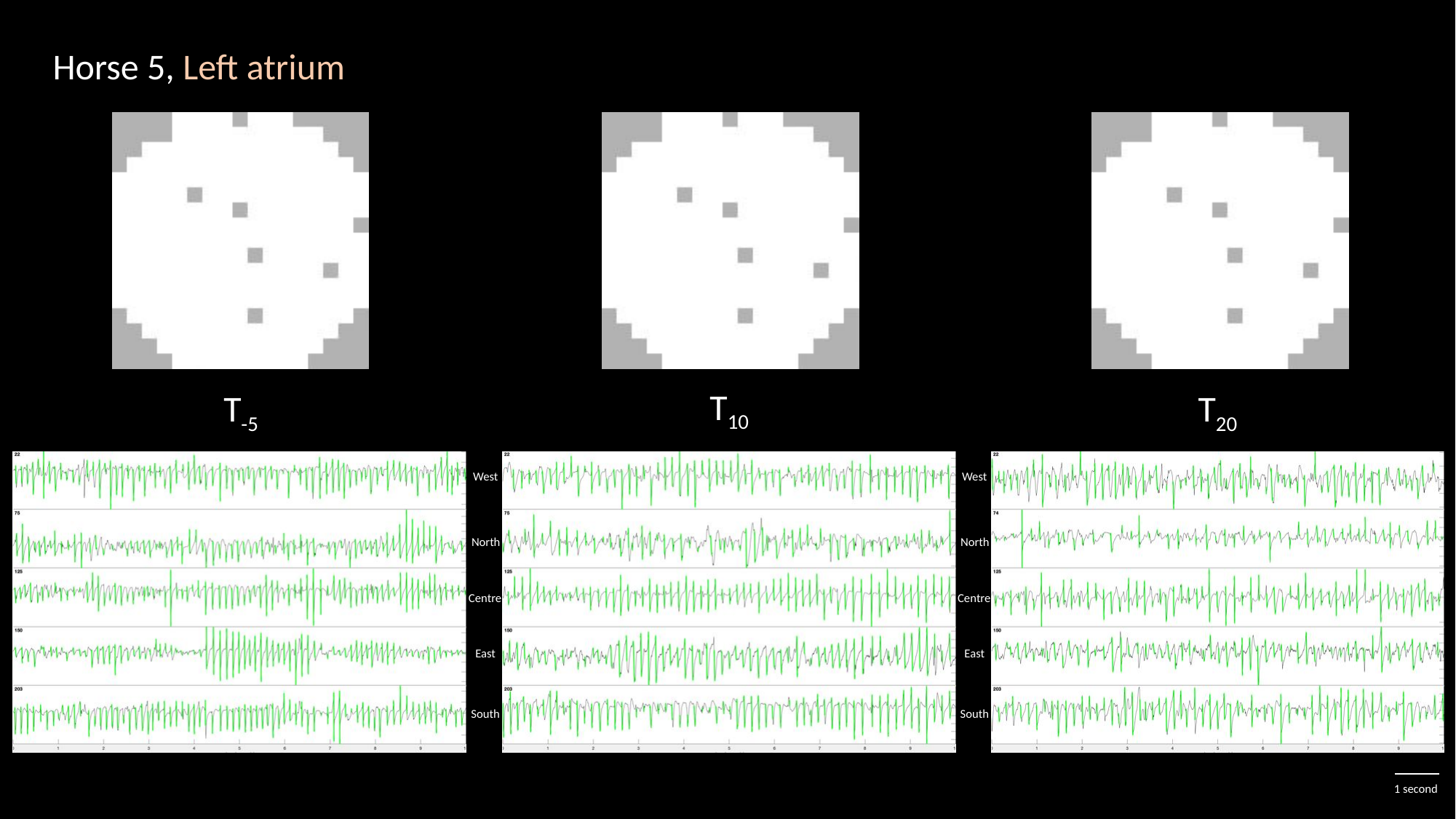

Horse 5, Left atrium
T10
T20
T-5
West
North
Centre
East
South
West
North
Centre
East
South
1 second

Supplement: Supplementary file 8 [file Presentation_7.PPTX]

## Slide 1
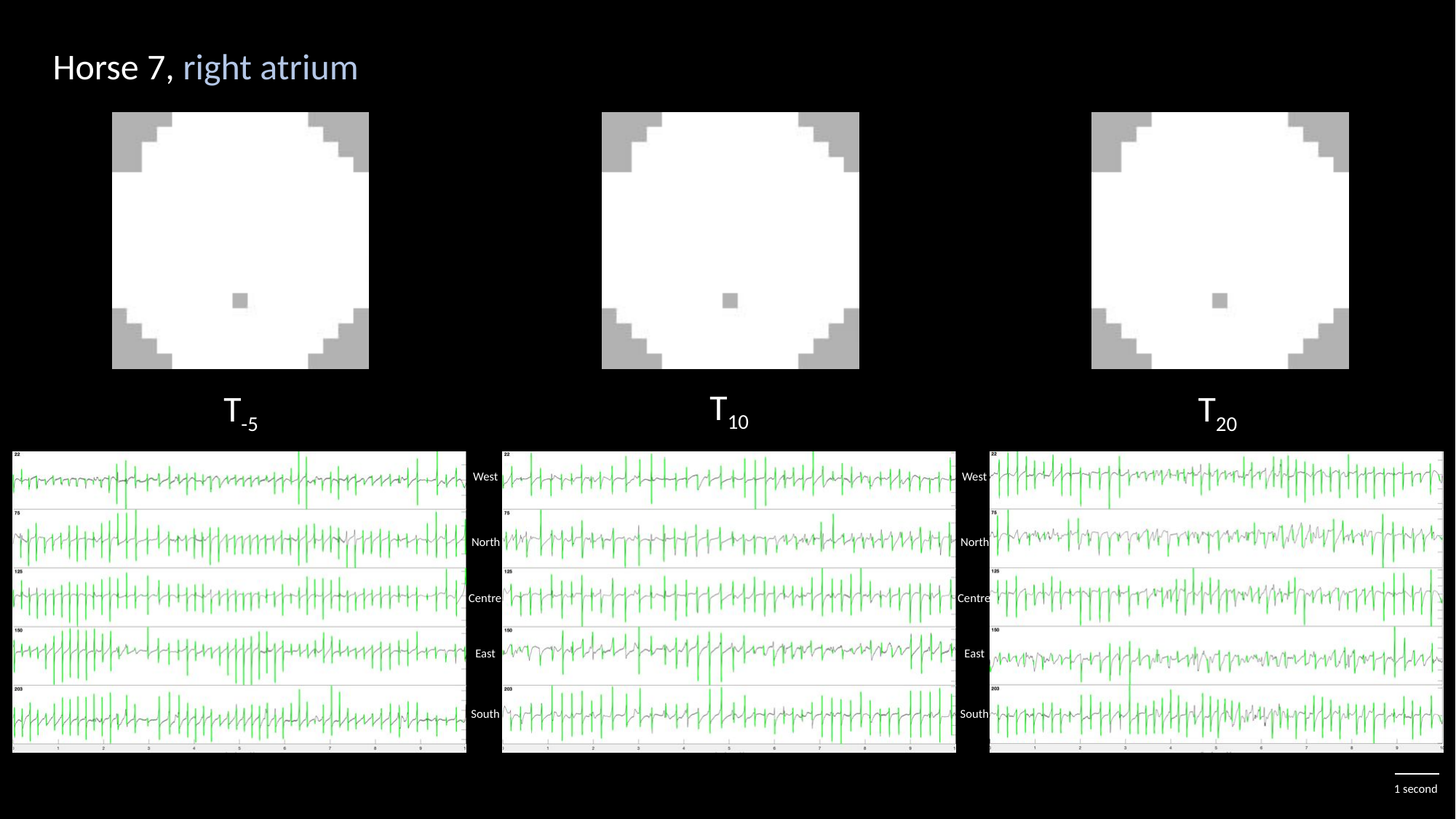

Horse 7, right atrium
T10
T20
T-5
West
North
Centre
East
South
West
North
Centre
East
South
1 second

Supplement: Supplementary file 9 [file Presentation_8.PPTX]

## Slide 1
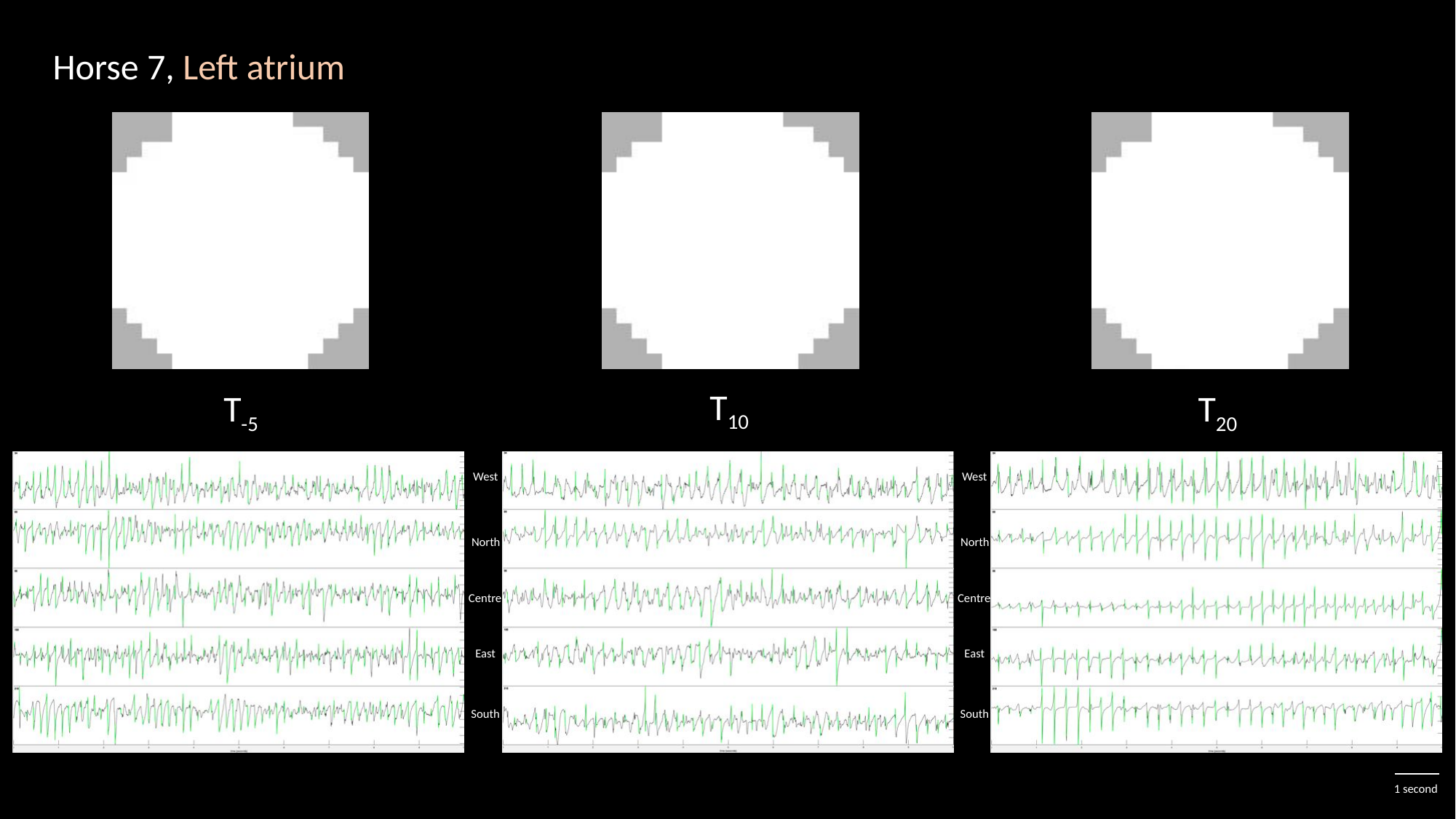

Horse 7, Left atrium
T10
T20
T-5
West
North
Centre
East
South
West
North
Centre
East
South
1 second

Supplement: Supplementary file 10 [file Presentation_9.PPTX]

## Slide 1
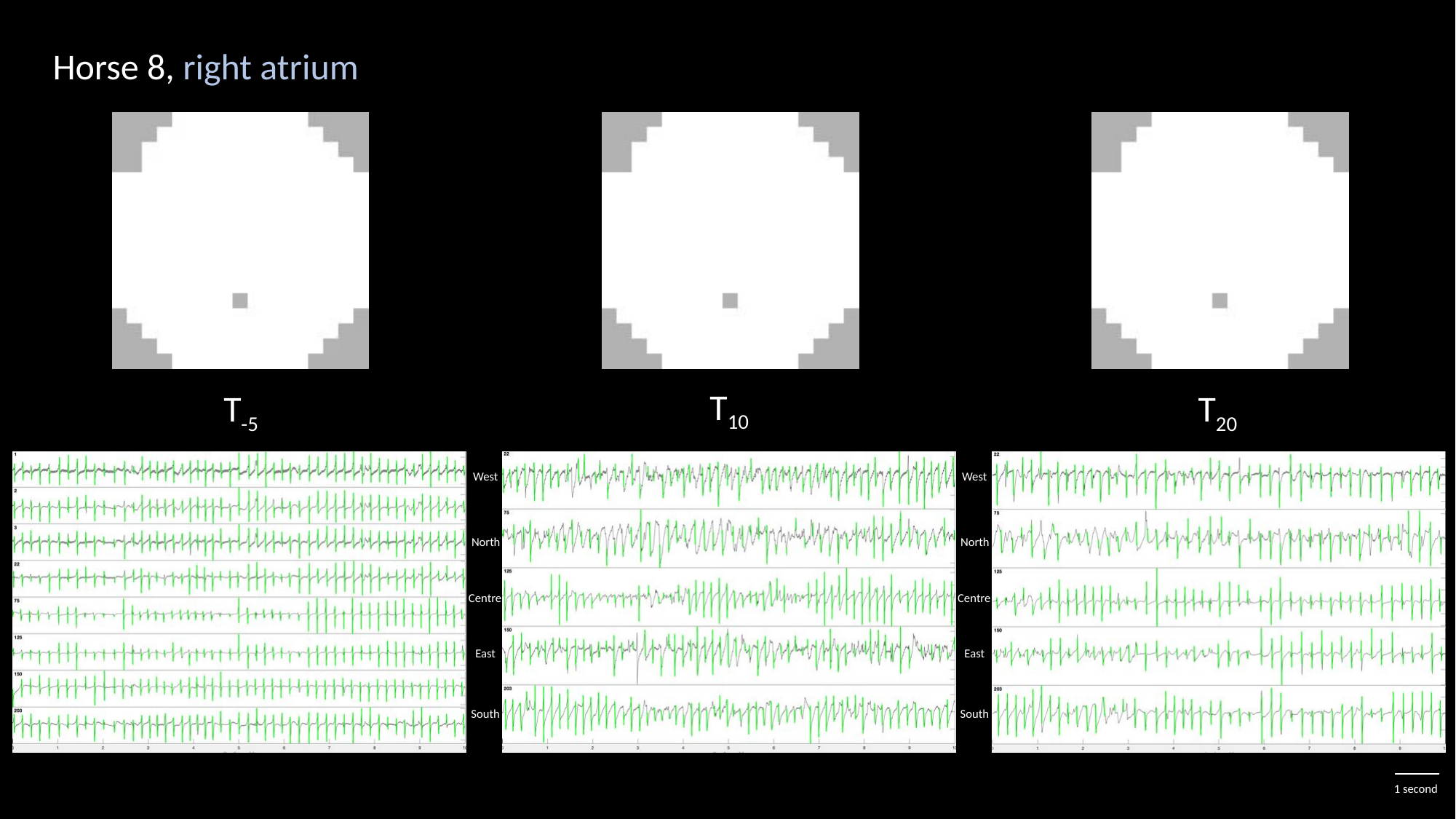

Horse 8, right atrium
T10
T20
T-5
West
North
Centre
East
South
West
North
Centre
East
South
1 second

Supplement: Supplementary file 11 [file Presentation_10.PPTX]

## Slide 1
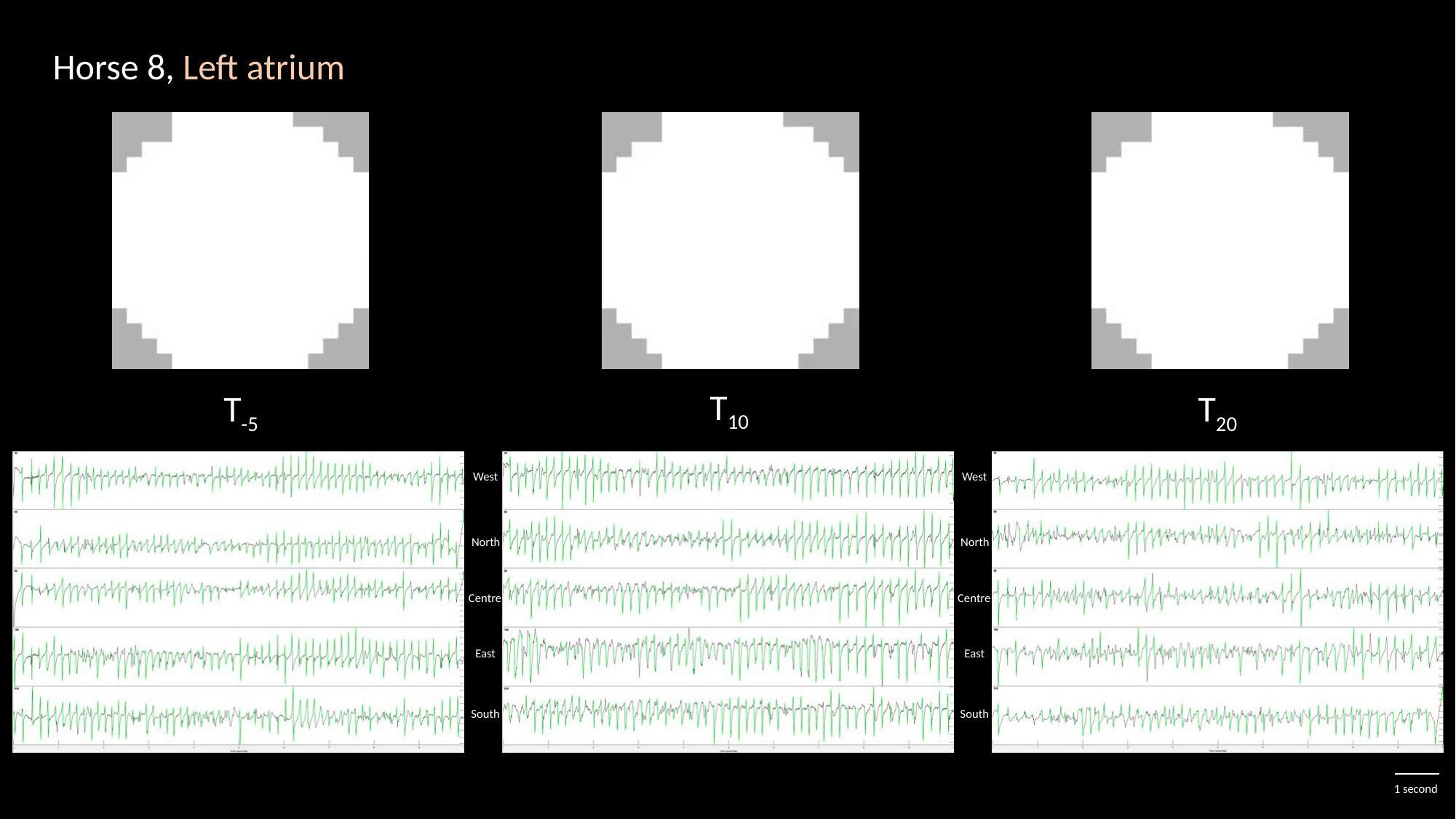

Horse 8, Left atrium
T10
T20
T-5
West
North
Centre
East
South
West
North
Centre
East
South
1 second

Supplement: Supplementary file 12 [file Presentation_11.PPTX]

## Slide 1
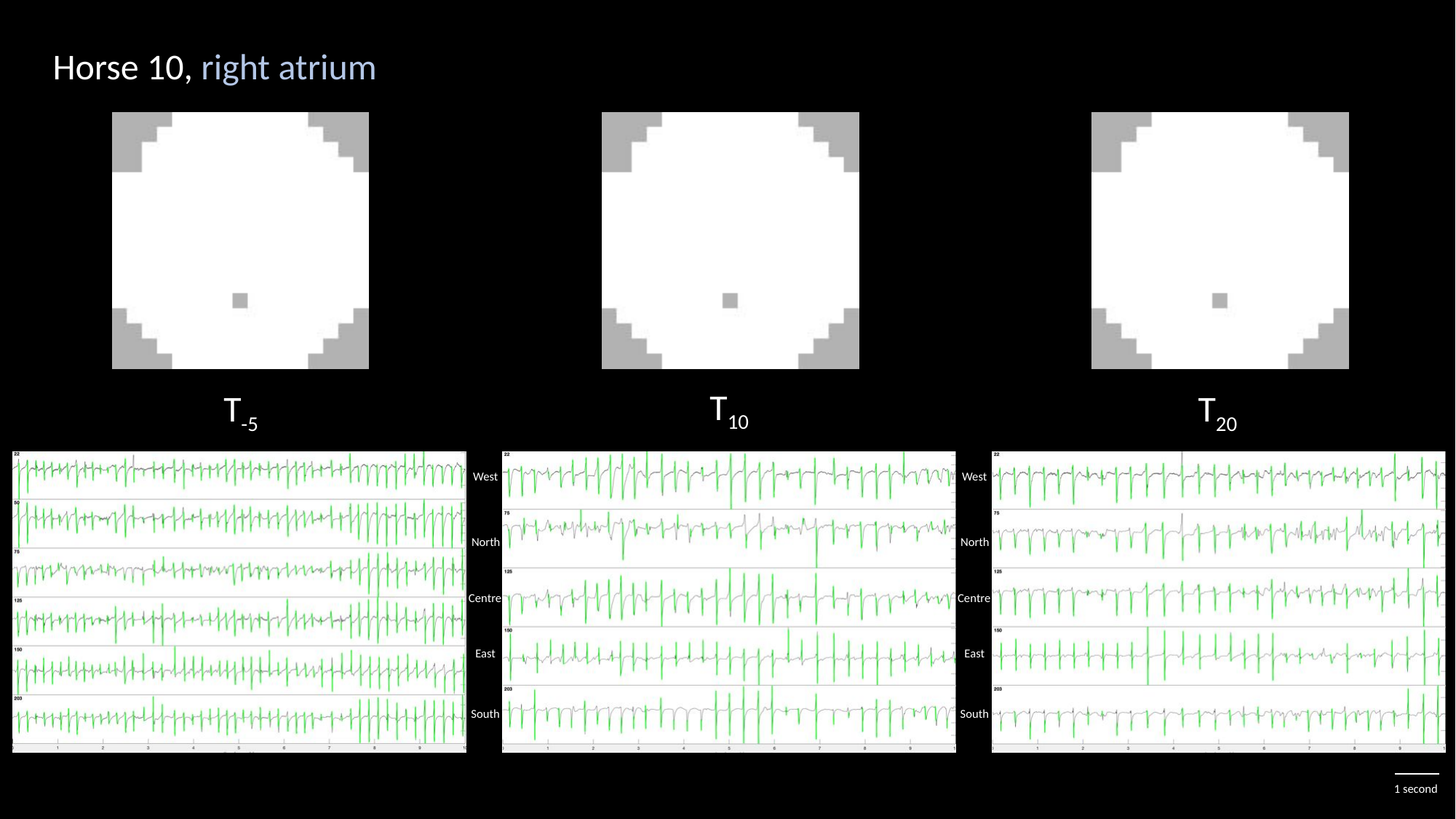

Horse 10, right atrium
T10
T20
T-5
West
North
Centre
East
South
West
North
Centre
East
South
1 second

Supplement: Supplementary file 13 [file Presentation_12.PPTX]

## Slide 1
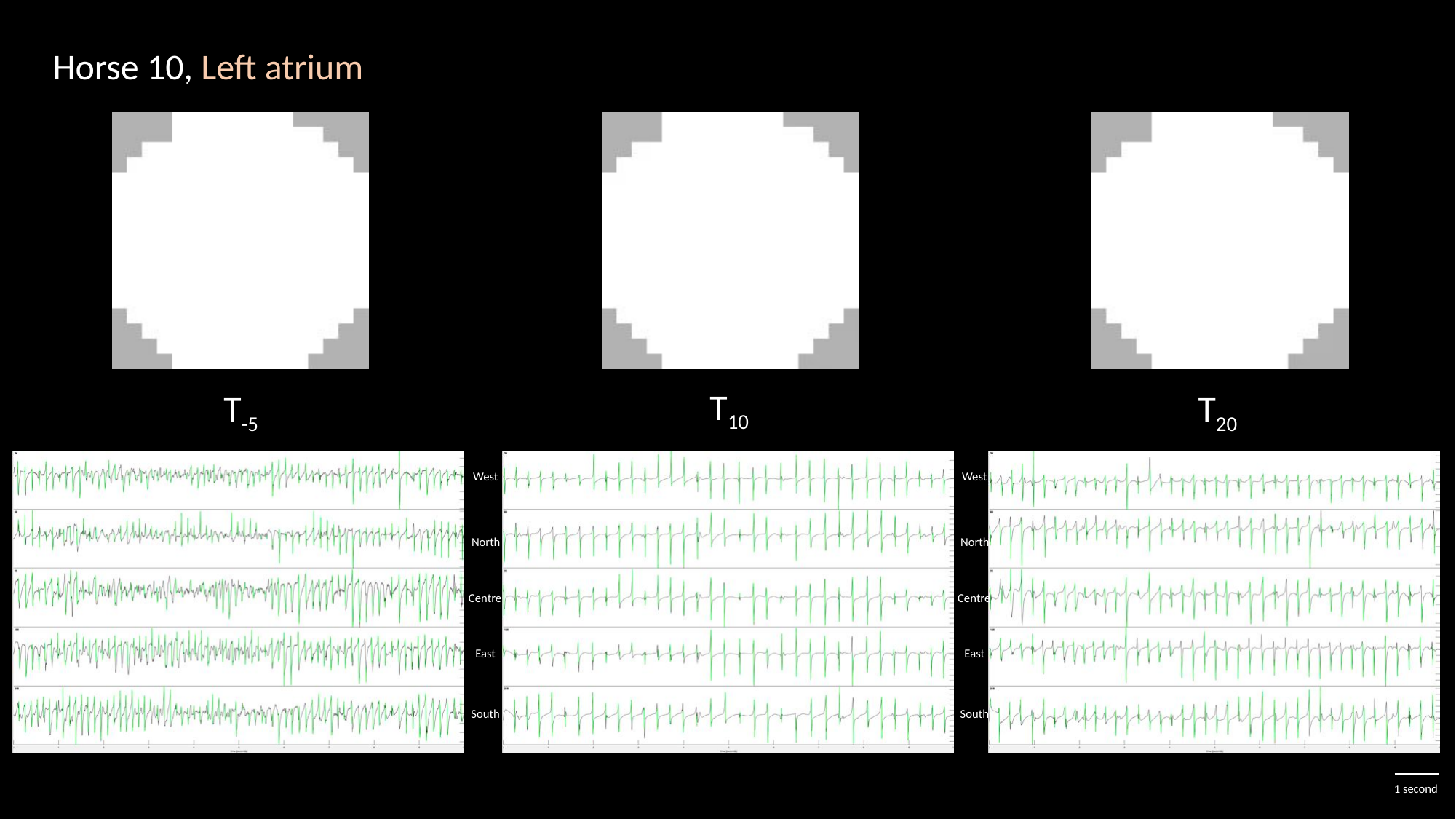

Horse 10, Left atrium
T10
T20
T-5
West
North
Centre
East
South
West
North
Centre
East
South
1 second

Supplement: Supplementary file 14 [file Presentation_13.PPTX]
